# A role for circular code properties in translation

**DOI:** 10.1038/s41598-021-87534-y

**Published:** 2021-04-28

**Authors:** Simone Giannerini, Diego Luis Gonzalez, Greta Goracci, Alberto Danielli

**Affiliations:** 1 Department of Statistical Sciences, University of Bologna, Bologna, 40126 Italy; 2 Department of Pharmacy and Biotechnology, University of Bologna, Bologna, 40126 Italy; 3 Institute for Microelectronics and Microsystems - Bologna Unit, CNR, Bologna, 40129 Italy

**Keywords:** Applied mathematics, Biophysics, Translation

## Abstract

Circular codes represent a form of coding allowing detection/correction of frame-shift errors. Building on recent theoretical advances on circular codes, we provide evidence that protein coding sequences exhibit in-frame circular code marks, that are absent in introns and are intimately linked to the keto-amino transformation of codon bases. These properties strongly correlate with translation speed, codon influence and protein synthesis levels. Strikingly, circular code marks are absent at the beginning of coding sequences, but stably occur 40 codons after the initiator codon, hinting at the translation elongation process. Finally, we use the lens of circular codes to show that codon influence on translation correlates with the strong-weak dichotomy of the first two bases of the codon. The results can lead to defining new universal tools for sequence indicators and sequence optimization for bioinformatics and biotechnological applications, and can shed light on the molecular mechanisms behind the decoding process.

## Introduction

The genetic code is nearly universal across all living organisms. Its degeneracy, mapping 64 three-letter codons to 20 amino acids and three stop codons, is highly conserved. This conservation has evolved to minimize the effects of genetic mutations and translational decoding errors, thus providing optimal robustness in the flow of the genetic information^1^. Moreover, the universal genetic code allows to tolerate arbitrary nucleotide sequences within protein-coding regions better than other possible codes. Such feature facilitates the inclusion of additional information beyond the coding of sequences of amino acids^2^. For example, deleterious effects of frame-shift translation errors can be reduced by increasing the probability of encountering stop codons out of frame; indeed, additional informational layers have been identified inside protein coding regions (see^3^ for a review on this topic). Nevertheless, many of the correlations between the information contained in the mRNA and different aspects of protein synthesis are still poorly understood. The term code is often used in an imprecise and descriptive manner without including the main features of a formal code, i.e., the identification of a definite coding and decoding strategy that can be represented symbolically. This is the case, in particular, for the correlations found between CUB (Codon Usage Bias) and protein synthesis rates.

The degeneracy of the genetic code enables to synthesize the same protein from a huge number of mRNA sequences encompassing synonymous codons. Growing evidence suggests that synonymous codons in coding sequences are not neutral with respect to the translation process, influencing the protein synthesis rates from bacteria to eukarya. It is generally accomplished that this codon bias contributes to the translation efficiency at the elongation step^4^, which in turn may affect the stability of the translated mRNA^5^. As such, codon preferences have been intensively studied, both for improved protein production yields in biotechnological settings as well as for codon-optimized gene design in synthetic biology and genetic engineering projects^6^.

The mechanistic effects of codon bias were initially attributed to slow translation of sets of rare codons^7^, implying the co-variance of codon usage frequency with the levels of matching tRNA pools and the deriving attenuation of translation elongation rates at infrequently used codons^8^. The recent introduction of genome-wide ribosome profiling studies has questioned this simplistic view, since the net ribosome elongation rates are apparently relatively constant and marginally affected by rare codon frequency^9, 10^. On the other hand, several studies correlated the effect of codon bias either with the stability of secondary structures at the 5$^{\prime }$ end of the mRNA^11–13^, or with the intracistronic occurrence of Shine-Dalgarno-like sequences mimicking the ribosome binding site^14^. Moreover, different types of codon bias have been described, including synonymous codon co-occurrence, allowing for rapid recycling of the exhaust tRNA in highly expressed genes^15^, or non-synonymous codon pair bias, dependent on optimal interactions of tRNAs in the A and P sites of the ribosome^4, 16, 17^.

An elegant study engineered a *his* operon leader peptide gene reporter in *E. coli* to investigate the local effects of codon context on in vivo translation speed^18^. Results demonstrated that the rate at which ribosomes translate individual synonymous codons varies considerably, and that the apparent speed at which a given codon is translated is influenced by flanking ones.

Recently, the codon influence on protein synthesis rates was assayed in greater depth, by integrating statistical analyses of large scale protein expression data sets with a systematic evaluation of local and global mRNA properties^5, 19, 20^. In particular, in^5^, a logistic regression model is used to build a codon-influence metric, validated by biochemical experiments, demonstrating that codon content is able to modulate the kinetic competition between translation elongation rates and mRNA stability. mRNA-folding effects generally prevail at the 5$^{\prime }$ end of the coding sequence^11^ and appear to be cumulatively weaker than codon bias effects^5^. Finally, it was shown that a major determinant of mRNA half-life and stability is the codon-optimized rate of translational elongation^21^.

Despite these advances, the theoretical principles behind the empirical effects of codon bias on translation efficiency remain poorly addressed. A possible correlative link between codon bias and reading frame maintenance was inferred from the statistical analysis of a large set of protein coding sequences in the three possible reading frames, resulting in the discovery that the set of most frequent in-frame codons formed a circular code^22–24^. In brief, a circular code is a special set of codons that allows to build sequences where the reading frames can be identified. This observation revived the study of protein expression from the point of view of coding theory initiated by Crick with the introduction of comma-free codes^25, 26^, and circular codes have been proposed as putative remnants of primeval comma-free codes^27, 28^. If genetic sequences formed with words/trinucleotides of a circular code are put on a circle and are read in the three possible reading frames, then there is only one reading frame that will contain only words from such code as the other reading frames will contain also words/trinucleotides that do not belong to such code (see Methods for more details on comma-free codes and a more formal definition of circular codes). Recent developments on the theory of circular codes led to postulate the existence of a coding strategy underlying the process of reading frame maintenance^29–32^. The circular code found in Arques and Michel^22^ belongs to a set of 216 codes possessing the following desirable properties: 1) they are *self complementary*: if a codon belongs to a code, then also its reverse complement belongs to the code; this property is important since it enables reading frame retrieval in both sense and antisense; 2) they are $C^3$: the circular permutations of the codons of a circular code also form a circular code; this property allows frame retrieval in all the three reading frames; 3) they are maximal: they contain 20 codons, which is the maximum allowed number for a trinucleotide circular code. This number result from the fact that periodic codons, (AAA, TTT, CCC, GGG), and circular permutations of a given codon, are forbidden because they break the circularity property. Thus, excluding the periodic codons we have $64-4= 60$ codons available to build a circular code in a given frame; moreover, for any chosen codon we need to exclude its two circular permutations (for example if CAT belongs to the code, we need to exclude both TCA and ATC); this leads to a maximal number of 20 codons, i.e., a code with 21 codons cannot be circular.

In the following we use the list of 216 codes given in^33^ and label them according to the order given there, so that we denote a generic *i*-th code of 20 codons with $X_{i}$, $i = 1,\dots ,216$. In^30^, it is shown that such set can be partitioned into 27 equivalence classes conforming to a group theoretic framework characterized by 8 nucleotide transformations that are isomorphic to the symmetries of the square, see Table 3. Table 4 shows an example of an equivalence class formed by 8 circular codes linked by such transformations. In practice, given a set of 8 codes that form a class, if one applies to them the 8 transformations of Table 3 then one always obtains a code of the same class. It has been postulated that this mathematical structure could be correlated with the correct transmission of information and frame maintenance during translation^29, 34^.

Such premises encouraged us to investigate more thoroughly whether circular codes can provide a theoretical framework able to explain or predict the effects of codon bias on translation. The key parameters we use to investigate the role of circular code properties on translation, is the **coverage** of a circular code over a specific sequence or organism. It is the cumulative codon usage of the set of codons belonging to that code and can be seen as a measure of its “compliance” with the coding sequence (see Methods and also Gonzalez et al.^29^). In order to explore the relationship of circular codes with extant coding sequences, we compare systematically the coverage of the 216 circular codes, partitioned in 27 equivalence classes, with the codon usage of a large set of organisms. Also, we re-analyze the results of three different experiments on translation efficiency.

## Results

### Circular codes’ coverage exhibits universal properties

We have analyzed the whole Codon Usage Database (https://www.kazusa.or.jp/codon/) to show the coverage (in percentage) for the 216 circular codes partitioned in 27 equivalence classes^30^. As a paradigmatic example we present the results for 8 codes forming the equivalence class of Table 4 (the results for the remaining classes are reported in the Supplementary Information). The results are presented in Table 1. As expected, each code has a distinct degree of coverage reflecting taxon-specific codon usage. For instance, code $X_{173}$ covers very well bacteria, i.e. the 46.4% of the codons of all bacterial genomes belong to code $X_{173}$. In contrast, the coverage for plants is lower (39.7%). Such disparity is reflected in the absolute ranks shown in the middle panel: for bacteria, code $X_{173}$ ranks 2nd among the 216 codes whereas for plants it ranks 16th. This heterogeneity is evident also for the other 7 codes of the class for all the kingdoms. However, if we consider the ranks of these coverages inside the equivalence class (lower panel), then a neat taxon-independent ordering among the 8 codes emerges, i.e. in this case, code $X_{173}$ is always the best of its class, code $X_{176}$ is always the second etc., irrespective of the species-specific codon usage. Surprisingly, this property holds for each of the 27 equivalence classes (see Table 2 of the Supplementary Information). Even more remarkably, the worst code within each class (code with the least coverage) invariably coincides with the chemical Keto-Amino transformation of the best one. In the example of Table 1, code $X_{173}$ is always the best code and its Keto-Amino transformation KM$(X_{173}) = X_{192}$ is always the worst within the class. This establishes an important link between the codon usage and the Keto-Amino (KM) chemical transformation that will be discussed below.

**Table 1:** Coverage (upper panel), absolute ranks (mid panel) and relative ranks (lower panel) for the equivalence class of 8 circular codes presented in Table 4. The universality of the results is clear if we consider the ranks within classes: for instance the coverage of code $X_{173}$ for bacteria is $46.4\%$ (upper panel). It is not the highest coverage among the 216 codes, indeed it is the second (mid panel). However, it is always the highest within its class (lower panel). This universal behaviour holds for the whole set of 216 codes partitioned in 27 equivalence classes.

Coverage	$X_{173}$	$X_{176}$	$X_{203}$	$X_{206}$	$X_{183}$	$X_{182}$	$X_{193}$	$X_{192}$
Bacteria	46.4	43.9	36.0	33.6	26.8	22.8	22.1	18.1
Animals	42.0	38.8	35.9	32.8	28.6	26.2	25.8	23.4
Viral	43.2	40.3	35.9	33.0	28.4	26.1	24.7	22.4
Plants	39.7	36.7	34.8	31.7	29.3	27.5	25.3	23.5

Absolute rank	$X_{173}$	$X_{176}$	$X_{203}$	$X_{206}$	$X_{183}$	$X_{182}$	$X_{193}$	$X_{192}$
Bacteria	2	11	58	81	155	189	195	212
Animals	2	19	43	84	148	180	187	208
Viral	2	18	53	84	148	176	190	209
Plants	16	35	55	98	140	165	190	208

Relative rank	$X_{173}$	$X_{176}$	$X_{203}$	$X_{206}$	$X_{183}$	$X_{182}$	$X_{193}$	$X_{192}$
Bacteria	1	2	3	4	5	6	7	8
Animals	1	2	3	4	5	6	7	8
Viral	1	2	3	4	5	6	7	8
Plants	1	2	3	4	5	6	7	8

To demonstrate that this property is not the trivial consequence of the fact that the more a set of codons is recurrent then, the less recurrent are codons that do not belong to the same set, we performed a bootstrap test, computed over 291 genomes with more than 1 million codons, to explore the relation between the coverage of the best and the worst code in an equivalence class. The data clearly show that within each equivalence class the best code does not transform into the worse code by sheer chance through the KM-transformation (see Supplementary Information, Sect. 2.1.1).

These results demonstrate that universal symmetry properties of coding sequences emerge when analyzed through the theoretical framework of circular codes, irrespectively of the species-specific codon-usage. Moreover, within each equivalence class, the Keto-Amino transformation of the code possessing the best coverage always leads to the worst covering code of the same class. Thus, a universal ordering structure, conserved across domains of life, emerges beyond the heterogeneity of species-specific codon usage.

### Universal frame marks in coding sequences

The biological functions associated with circular code properties are basically unexplored. These properties may be explained as a fossilized memory of comma-free (self-synchronizable) coding in primeval forms of life^28^, or tentatively associated with reading frame maintenance during protein synthesis^35^. Thus, to explore whether the universal ranking property shown above, is valid also out of frame, we extended the analysis of the coverage of circular codes to the three reading frames of coding sequences for 25 well-annotated eukaryotic species (Table 3 of the Supplementary Information). The results are shown in Table 4-9 of the Supplementary Information. Remarkably, despite the variability of the codon usage among the different species, the ranking within each equivalence class is always preserved in the three frames. For example, for frame + 1, Tables 6-7 (+2, Tables 8-9) of the Supplementary Information, the first (second) circular permutation of the best codes has always the highest coverage, whereas their keto-amino transformation always leads to the worst covering codes within their equivalence class. We argue that this universal property observed in all the reading frames is connected to the circularity property of the codes. In order to show this we have generated 1000 random codes such that *i*) they are self complementary, *ii*) they are maximal, *iii*) they do not contain stop codons or periodic codons (AAA, TTT, CCC, GGG), *iv*) they are **not** circular. Overall, for each frame, we obtain 8000 codes by applying the transformations of the dihedral group to the 1000 random codes. The hypothesis that the structure observed can be generated by random codes is rejected with a *p*-value of $3.9\times 10^{-8}$ (see the Supplementary Information, Sect. 2.2 for more details).

**Figure 1:**
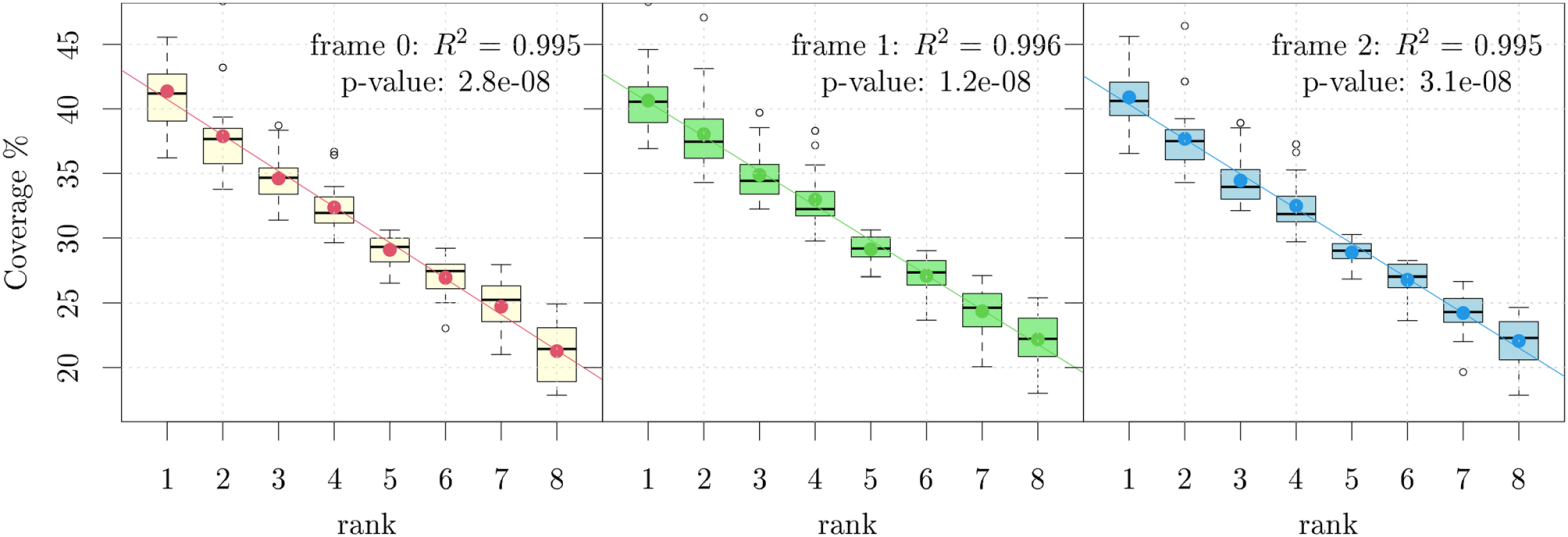
Universal scaling properties of the coverage within equivalence classes for the three reading frames.

When ordered through the ranks, the coverage shows a strong linear scaling. This is shown in Fig. 1(left) that reports the boxplots of the coverage (percent) of the 8 circular codes of Table 4 over the in-frame coding sequences of the 25 eukaryotic genomes analysed. The same linear scaling is observed for the coverage of the first and second circularly permuted codes, over the same coding sequences read out-of-frame +1 (central panel) and +2 (right panel), respectively. Scaling laws are important in Information Theory^36^, in Statistical Mechanics and in Dynamical System Theory^37^ and have also been associated to universal properties and long range correlations in DNA^38^. Intriguingly, the structure uncovered in coding sequences is completely absent in introns (Table 10 of the Supplementary Information).

In conclusion, each circular code has a distinct degree of coverage with respect to the species-specific codon usage of different organisms. Notably, however, behind this variability we observed universal properties, linking the coverage inside equivalence classes with the set of transformations of the codons of the codes. Such strong organization is present in coding sequences but not in introns.

### Circular codes and in vivo translation speed

The distinctive organization uncovered above, present in the three frames of coding sequences and absent in intron sequences, hints at a biological role in the translation process. We explored this possibility by analysing the single codon global translation speeds resulting from an *E. coli his* operon attenuator reporter system^18^. In this system, higher transcription rates of the reporter correspond to lower translation speeds.

**Figure 2:**
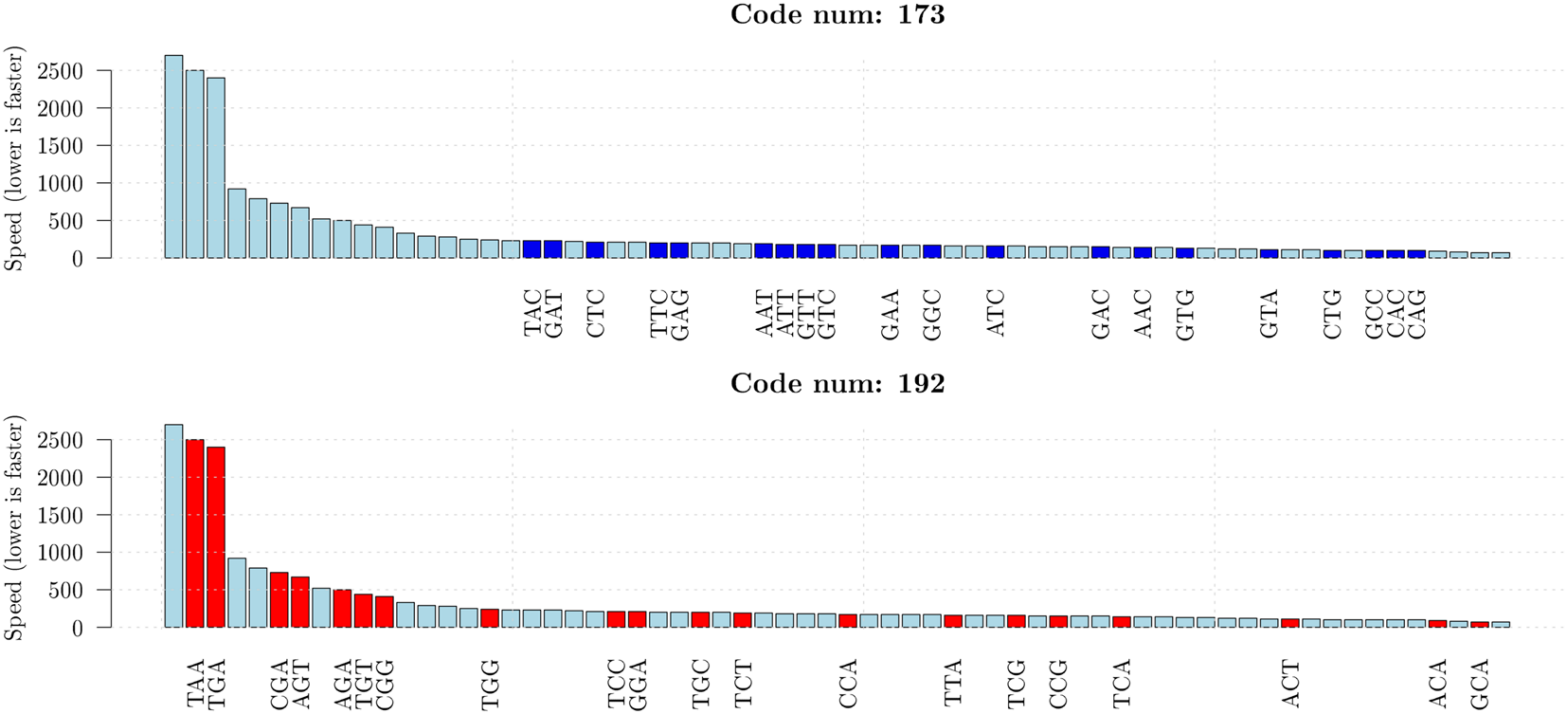
Ordered speed of the 64 codons, the data come from the experiment of^18^ and lower values indicate faster codons. The codons coloured in blue (upper panel) and in red (lower panel) belong to code $X_{173}$ and $X_{192}$, respectively. They are the best and worst codes within the set of 8 codes forming the equivalence class shown in Table 4.

**Figure 3:**
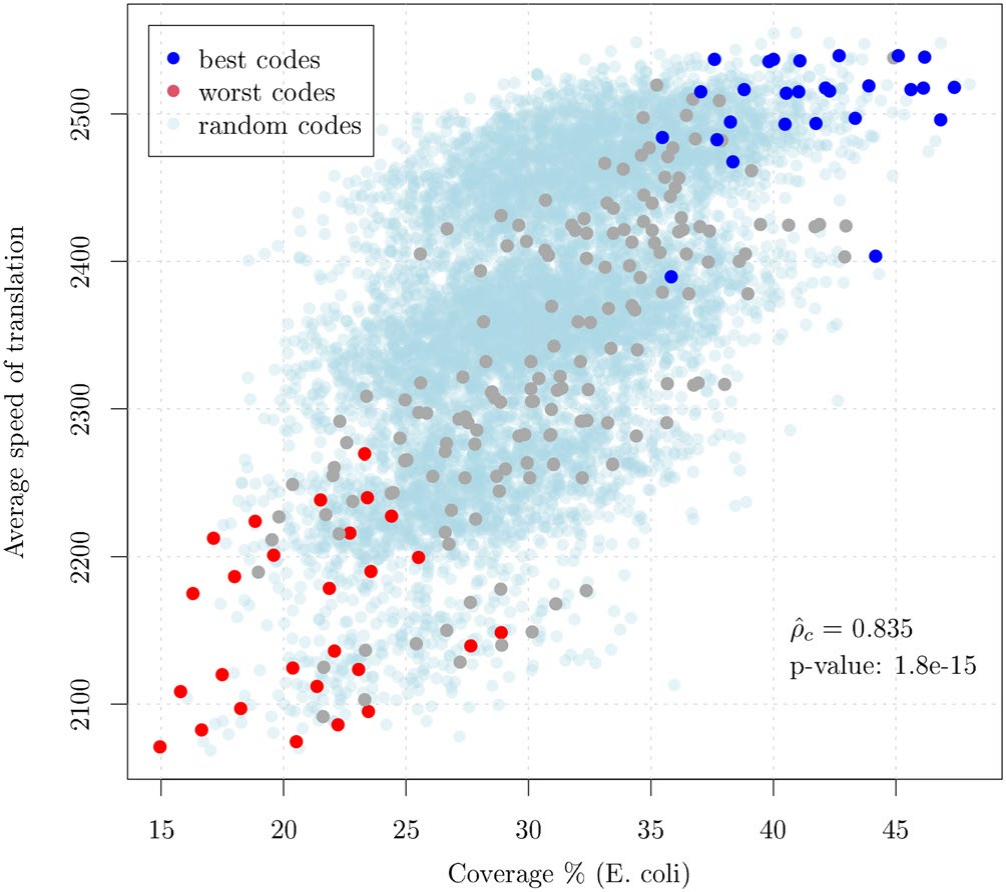
Average speed of translation versus Coverage (percent) for the 216 circular codes partitioned in 27 equivalence classes of 8 codes each. The points in blue and red correspond to the 27 best and 27 worst codes (corresponding to the KM transformation of the best codes) within their associated equivalence class. Clearly, the coverage is a predictor of the speed of translation and the best and worst codes within their equivalence class clusterize. The results for 8000 random codes are also shown in light blue and the *p*-value for the test that the observed correlation is equal to that of random codes is reported.

Remarkably, all the codons of the best code $X_{173}$ fall within the set of fast translated codons, whereas the great part of the codons of code $X_{192}$ appears to be among the slowest (see Fig. 2). In order to verify whether this property holds for all the 27 equivalence classes we have computed the average speed for each code (i.e. the average speed of the set of 20 codons that compose each code) as a function of the coverage of the code in *E. coli* (i.e. the cumulative codon usage of the 20 codons of each code). Figure 3 shows the average speed versus the coverage for the 216 circular codes, where we have marked in blue the 27 codes that rank first within their equivalence class and in red the 27 codes that rank last. The results for the 8000 random codes described above are also shown (in light blue). In order to enhance the comprehension we have reversed the scale so that higher values correspond to higher speeds. The best and worst codes form clusters that contain the fastest and the slowest codes, respectively. As mentioned above, the two sets are related by the chemical KM transformation. The relationship between circular-code-coverage and speed of translation appears to be linear with a correlation coefficient ${\hat{\rho _c}} =0.835$. From the plot, it appears that the 216 codes and random codes belong to different probability distributions and this is particularly evident for some of the best codes. We have tested the significance of the result by comparing the above correlation coefficient with that obtained from the 8000 random codes. The null hypothesis of equality is rejected with a *p*-value equal to 1.8e−15. The details of the test are reported in the Supplementary Information. Overall, the results would indicate that the coverage of a circular code can be a predictor of the speed of translation.

### Circular codes and codon influence on protein expression

In order to further establish a link between circular codes theory and protein synthesis we analyzed the experimental evidence reported in^5^ where the authors use a black box logistic regression model over a large-scale protein expression dataset. Their aim was to assess the influence on protein synthesis of both mRNA sequence parameters and single codons. After accounting for sequence parameters such as predicted free folding energy or head folding indicators, they found a significant effect of individual codons that appears several positions after the initiator codon and stabilizes after about 16 codons. Conveniently, this analysis does not suffer from the presence of stop codons in the codes that may bias the average translation speed presented in Fig. 3.

**Figure 4:**
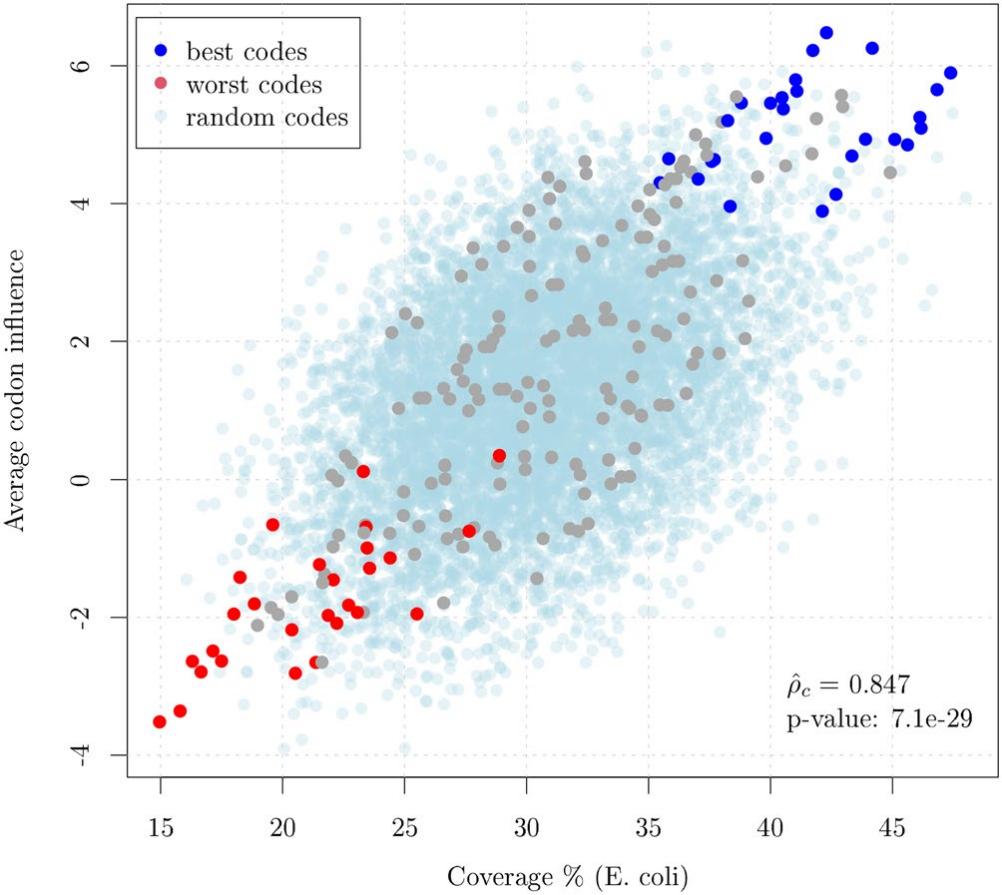
Average codon influence versus Coverage (percent) computed on the 216 circular codes partitioned in 27 equivalence classes of 8 codes each. The points in blue and red correspond to the 27 best and 27 worst codes within their associated equivalence class, respectively. As for the speed of translation (Fig. 3), the coverage is a predictor of codon influence and the best and worst codes within their equivalence class clusterize. The results for 8000 random codes are also shown in light blue and the *p*-value for the test that the observed correlation is equal to that of random codes is reported.

Consistently with the codon speed reported in the previous section, the codon influence is strongly correlated with the circular code coverage (${\hat{\rho _c}}=0.847$, Fig. 4). Also in this case, we have superimposed the results obtained from the aforementioned 8000 random codes and tested the equality of the corresponding correlation coefficients. The null hypothesis is rejected with a *p*-value = 7.1e−29. Notice that this correlation cannot be explained in terms of single codon usage as there is no evident correlation between single codon influence and single codon usage (Fig. 2 of the Supplementary Information).

### Circular code properties and ribosome residence Time

**Figure 5:**
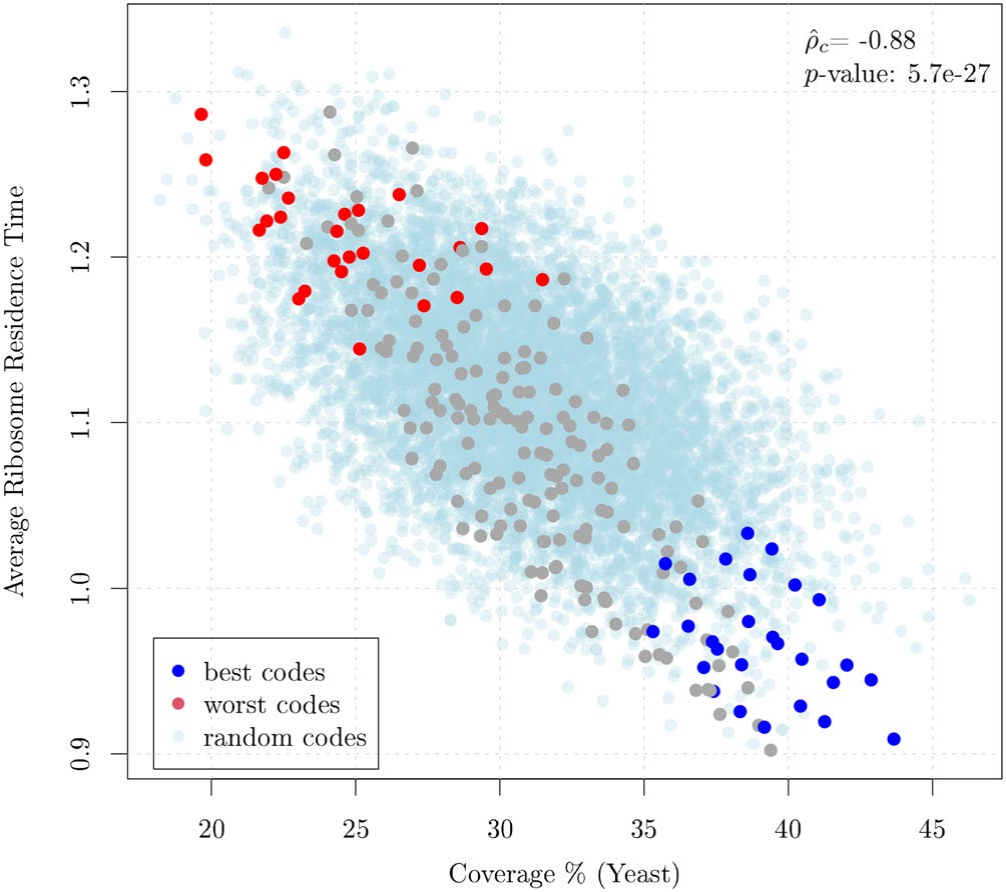
Average Ribosome Residence Time (RRT) versus Coverage (percent) computed on the 216 circular codes partitioned in 27 equivalence classes of 8 codes each. The points in blue and red correspond to the 27 best and 27 worst codes within their associated equivalence class, respectively. Similarly to the experiments shown above, the coverage is a predictor of the RRT and the best and worst codes within their equivalence class clusterize. The results for 8000 random codes are also shown in light blue and the *p*-value for the test that the observed correlation is equal to that of random codes is reported.

To understand whether the correlation of circular code properties with translation are present in eukaryotes, we computed the predicted Ribosome Residence Time (RRT) of circular codes in yeast, using the codon data ribosome profiling experiments^19^, and compared these values, reflecting extant in vivo translation metrics, with the percent coverage (in yeast) of the circular codes. The ribosome profiling data clearly confirm the results obtained in the previous sections. Indeed, the Ribosome Residence Time is strongly correlated with the circular code coverage (${\hat{\rho _c}}=-0.88$, Fig. 5). As before, the light blue dots are obtained from the above 8000 random codes and we tested the equality of the correlation coefficients observed in random codes with that of circular codes. The null hypothesis is strongly rejected with a *p*-value = 5.7e−27. The correlation observed in random codes is similar to that of single codons (Fig. 3 of the Supplementary Information) and can be explained as such. These results neatly show that the correlation between circular code coverage and codon influence on protein expression holds true also in eukaryotes, hinting at conserved properties.

### Circular code codons are less abundant in the mRNA 5$^{\prime }$-head and 3$^{\prime }$-tail sequences

Several independent reports demonstrated that the folding energy at the 5$^{\prime }$ end of the mRNA explains most of the variation in protein expression levels, indicating that tightly folded messengers, obstructing the 30 nt ribosome binding site centered on the initiator codon, strongly influence translation initiation rates^11, 13, 20^. In^5^ it is shown that, by computing the increase in the likelihood ratio when adding to the model terms corresponding to the average value of the codon influence over rolling windows of 5, 10 and 15 codons, the influence of codons is enhanced in the first part of the sequence, especially from codon 7 to codon 16 and stabilizes after 32–35 codons.

**Figure 6:**
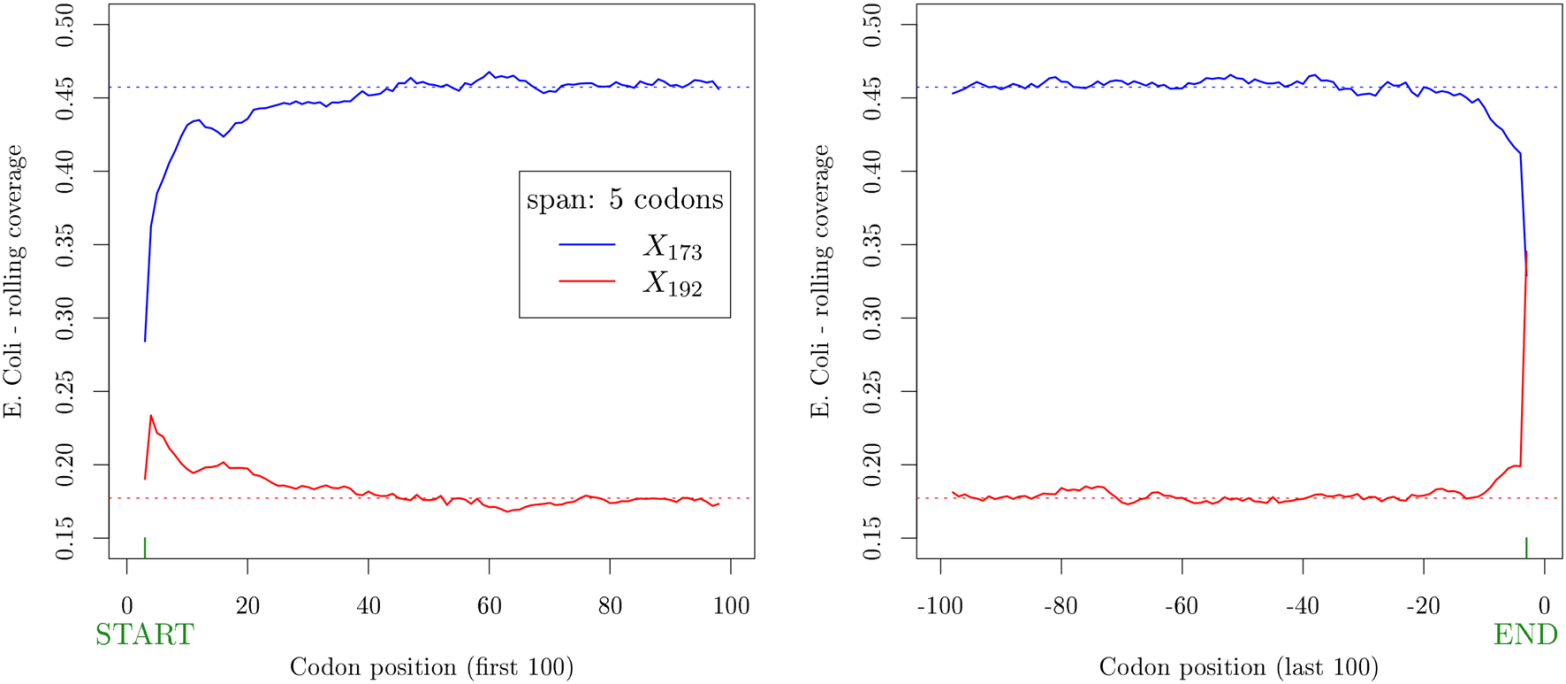
Rolling coverage (span: 5 codons) computed on the first (left) and last (right) 100 codon positions, averaged over the whole set of 3983 complete coding sequences of *E. coli*. The blue and red solid lines correspond to code $X_{173}$ and $X_{192}$, respectively. The dotted lines correspond to the global coverage of the codes over the whole genome.

If circular code properties play a role in translation, then we could expect a different coverage as a function of the position along the coding sequence. In Fig. 6(left) we plotted the coverage of codes $X_{173}$ (blue solid line) and $X_{192}$ (red solid line) over rolling windows of 5 codons, computed over the first 100 codons of each complete coding sequence of *E.coli*. Remarkably, both for code $X_{173}$ and $X_{192}$ there is a transient initial span (around 40 codon positions) after which the rolling coverage over 5 codons reaches the value of the global coverage over the entire genome and slightly fluctuates around it. While for code $X_{173}$ the rolling coverage for the first positions is always lower than the global coverage, the rolling coverage for code $X_{192}$ starts at a higher level with respect to the global coverage and decreases towards it. This appears to be a universal feature shared by all the organisms (see the Supplementary Information). The same is true for rolling windows up to 30 codons with no significant differences. The effect of the total codon content in the 3$^{\prime }$ tail of the mRNA sequence was also reported to be influential on expression^5^. Accordingly, we also observed a tail effect in the coverage of coding sequences (Fig. 6(right) and Supplementary Information).

These results indicate a lower coverage of the best circular code both in the head and in the tail of coding sequences, consistent with growing experimental evidence that other factors, such as mRNA folding energy, may predominate in those regions.

**Figure 7:**
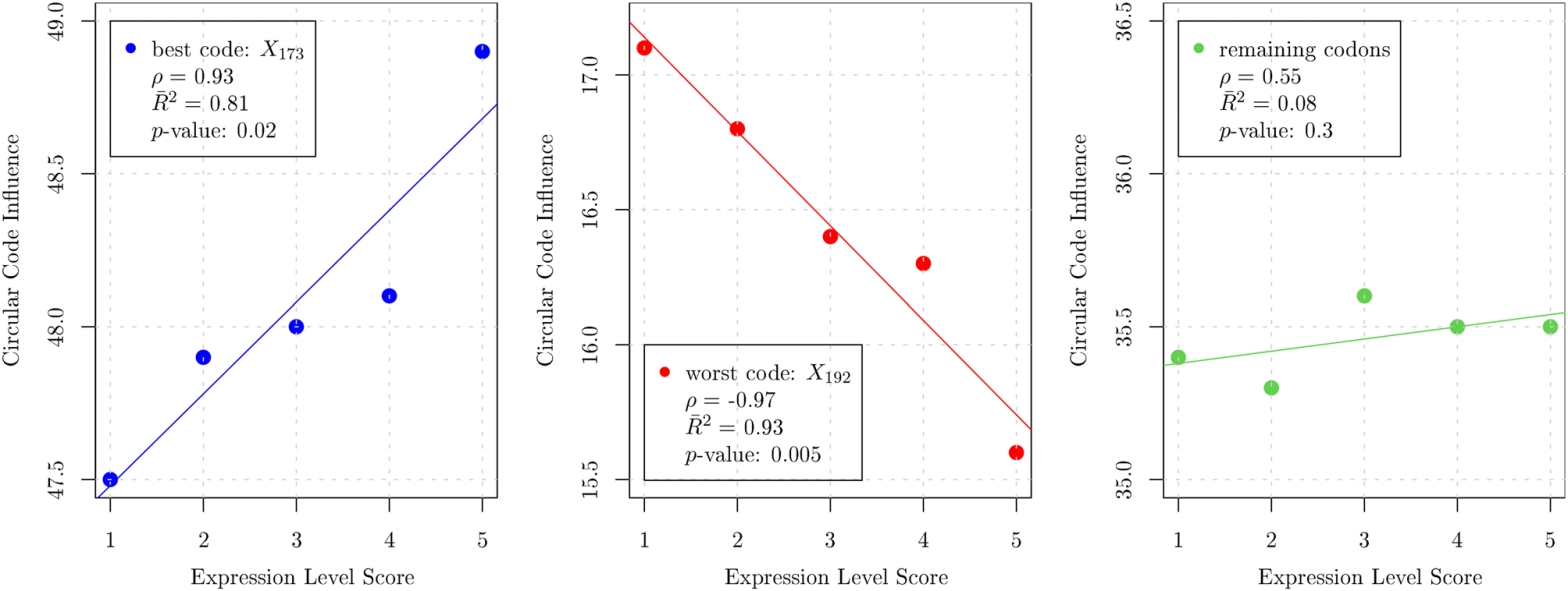
Expression level score versus average cumulative influence of circular codes. Left panel: best code $X_{173}$. Center panel: worst code $X_{192}$. Right panel: remaining codons, excluding stops.

### Protein synthesis levels correlate with circular code properties

To further explore the existence of a link between gene expression levels and circular codes, we computed the influence of each codon in a given sequence as the usage of such codons weighted by their specific influence. We analysed the set of 6348 sequences for which the protein synthesis levels had been previously measured and categorised, from 1 (low) to 5 (high)^5^. This enabled us to correlate the expression level with the average cumulative influence of codons belonging to the best and worst codes (Fig. 7). Clearly, a strong positive correlation ($\rho =0.93$) between expression levels and the influence of the best code emerges. Moreover, a strong negative correlation links the influence of the worst code to protein synthesis levels ($\rho =-0.97$). Even more remarkably, the remaining codons (the 21 codons that do not belong to either of the two former codes) fail to show any noticeable correlation, so that, on average, an increase in the expression level score is linked to an increase of the circular code influence for the best code and a corresponding decrease for the worst code. In this way, a clear link between circular code properties and protein synthesis levels has been established, pointing to the existence of a role played by circular code properties in translation. As such, we anticipate that circular code theory can be important for the optimization of gene sequences for the production of recombinant proteins.

### Circular code properties correlate with the S/W character of the first two nucleotides of the codon

Within each equivalence class the KM transformation always corresponds to passing from the best to the worst code, both in terms of coverage and translation efficiency, in agreement with recent experimental evidence of a correlation between codon usage and rate of decoding^19^. In the KM transformation, keto (K; T or G) is transformed into amino (M; C or A) and viceversa (T$\leftrightarrow$C, G$\leftrightarrow$A). This invariably changes the character of the base from strong (S; G or C) to weak (W; A or T), and this transformation appears to accompany remarkable effects on translation. Our results therefore indicate that the molecular biology in the decoding process may be significantly affected by the S/W character of the codon bases. Indeed, it has been reported that AT-rich codons are decoded slightly faster than GC-rich codons^5, 19^. AT-rich codons result in weaker secondary structures in mRNAs and therefore in higher translation initiation rates^13^. However, at the elongation level a mechanistic explanation for faster decoding of AT-rich codons is still missing to date.

**Figure 8:**
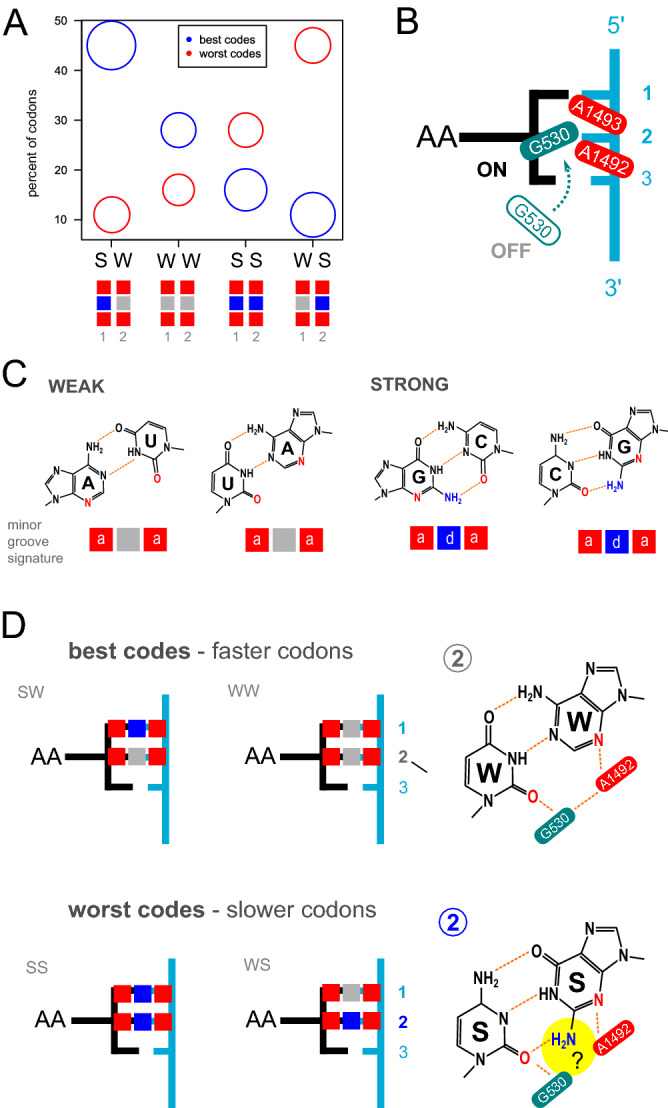
Correlation of circular code properties with the S/W character of the codon hint at the decoding mechanism dynamics. (**A**) Comparison of codon composition of best codes (blue) and worst codes (red) according to the S/W chemical dichotomy of the first two nucleotides of the codon. The area of the bubbles is proportional to the average codon influence. Codons of the kind SWN and WWN identify the best codes, i.e. those associated to a higher expression level and coverage. Conversely, codons of the kind SSN and WSN characterize the codes having lower expression level and coverage. Boxed rectangles depict H-bond pattern formation in the minor groove of the codon-anticodon minihelix in positions 1 and 2 (see panel C for molecular details). (**B**) Model for the cognate codon-anticodon recognition in the minor groove of the decoding center (A site) of the ribosome; mRNA (light blue), aminoacyl-tRNA (black), universally conserved A1492 and A1493 nucleotides forming A-minor motif (red); universally conserved G530 nucleotide involved in latch closure and H-bonding with A1492 and A1493 (petrol green); numbers denote the nucleotide position of the codon. (**C**) Molecular detail of all possible cognate Watson-Crick base pairs. The minor grove locates beneath each pair. Notice that W–W (A–U and U–A, respectively) and S–S (G–C and C–G) display the same signature of H-bond acceptors (a, red) and donors (d, blue). (**D**) Projection of the H-bond acceptor and donor signatures on the decoding center model. Faster codons (more abundant in better codes) lack the central H-bond donor in the second nucleotide of the codon (highlighted in yellow), which characterize the slowest codons (more frequent in worst codes). Note that the keto-amino (KM) transformation, invariably leading from the best to the worst code in each equivalence class, always transforms a W base in S and viceversa.

In this respect, a fascinating feature emerging from the analysis of the best and worst codes (e.g. $X_{173}$ and $X_{192}$, respectively), concerns the chemical nature of the bases of the first two nucleotides in the codon (Table 2). All the most influential codons of code $X_{173}$ are of the kind SWN (strong-weak-any), the remaining ones being of the kind WWN (weak-weak-any). Conversely, by virtue of the KM transformation linking the two codes, the codons of code $X_{192}$ are of the kind WSN or SSN. On average, these codons appear to be less influential. Hence, we investigated whether this property holds also for the remaining codes. We computed the average frequencies of SWN, WWN, SSN and WSN codons for the group of best codes (blue) and worst codes (red), see Fig. 8, panel A, where the area of the bubbles is proportional to the average influence of each group of codons. Clearly, codons of the kind SWN and WWN identify the best codes, i.e. those associated to a higher expression level and coverage. Conversely, codons of the kind SSN and WSN characterize the codes having lower expression level and coverage.

Thus, the analysis of circular code properties appears to point at a link between the S/W dichotomy in the first two bases of the codon and translation levels. In particular, the results indicate a codon ordering where SWN codons confer the highest expression levels. In this respect, the theory of circular codes allowed to uncover the possible role played by the S/W dichotomy in the dynamics of the decoding process. Strikingly, this feature can be linked to the molecular interactions taking place in the ribosome decoding center (see below).

## Discussion

We have shown that circular codes theory provides a new and powerful key to understanding the influence of codon bias on gene expression. Circular code coverage exhibits taxon-independent universal properties with a strong hierarchical organization. Independently from codon usage, universal frame marks are present in coding sequences and are absent in introns. Indeed, there are recurring properties, linking the coverage inside equivalence classes with the set of transformations of the codons of the codes. These properties strongly correlate with translation speed, codon influence and protein synthesis level. In accordance with the predominant effect of the secondary structure of mRNAs in the 5$^{\prime }$ ends on translation, circular code properties are absent at the beginning of coding sequences. Indeed, protein synthesis rates are governed by different mechanisms in the various regions of the mRNA. Generally, protein synthesis rates are mainly controlled at the assembly step of translation initiation factors on the 5$^{\prime }$UTR . The secondary structures formed by the 5$^{\prime }$UTR with the head region of the mRNA coding sequence have a paramount regulatory role in this process^11–13^. Accordingly, in the head region, the molecular determinants underlying protein synthesis rates are not linked to translation elongation, at least not for the first 30 nts of the head region which homes the footprint of the initiating ribosome. Beyond the head region, protein synthesis rates are governed principally by elongation dynamics^5^. Finally, in the tail region, encompassing the 30 nt footprint of the ribosome halted at the stop codon, the translation termination processes (release factors) determine the rate of polypeptide release, and thus protein synthesis. It is therefore interesting to notice that circular code coverage is absent in head and tail regions and only persists within the central part of the mRNA where translation elongation dictates the protein synthesis rate. In particular, the strength of circular code coverage is low for the first 40 codons of the transcript, where several other factors (recently reviewed in^3^), prevail in dictating the rate of initiation and early elongation. This observation strongly supports that compliance to circular code coverage is not linked to coding sequences *per se*, but interestingly, and more specifically, to a precise phase of translation (elongation) that is less affected by initiation factors, and for which less conceptualization has been provided to date. The results support the emerging idea^3^ that ORFs, in addition to UTRs, are populated with overlapping layers of information, e.g. synonymous codon usage, ribosomal frameshifting and mRNA stabilization marks etc, and that this ORF-encoded information is able to modulate translation speed or accuracy and thereby protein synthesis or folding rates. Another indication that circular code properties may be mainly involved with translation dynamics is the recent discovery of circular code periodicities in certain regions of the 16S rRNA. These findings have been tentatively explained with the need for correct frame synchronization and retrieval in primeval forms of the translation machinery^35, 39^.

In *E.coli* codon influence on protein expression correlates also with transcript stability and mRNA half-life^5^. It was postulated that codon bias modulates the kinetic competition between protein elongation and mRNA decay. We have therefore investigated whether codon-dependent mRNA stability could correlate with circular code properties. Results indicate that the codon stabilization coefficient (CSC), a codon metric derived from the correlation between codon frequency in transcripts and mRNA half-life experimental data, well matches with universal circular code properties (see Supplementary Information Fig.  7). As for the codon influence, this correlation is not evident at the level of single codons (see Supplementary Fig.  8). This finding is consistent with observations that codon optimality is a major determinant of mRNA stability both in bacteria as well as in eukaryotes^17, 40^, even if some heterogeneity in the sensitivity towards translation rates may occur between different mRNAs.

Perhaps the most interesting observation gathered through the lens of circular codes is that their coverage correlates not only with protein synthesis rates but also with the S/W dichotomy in the first two nucleotides of codons. From a mechanistic point of view, an exact Watson-Crick base-pairing between codon and anticodon in the first two codon positions is indispensable for the correct (cognate) decoding in the A-site of the ribosome^16, 41^. Functional and structural evidences indicate that during the decoding process universally conserved bases of the 16S rRNA closely interact with the codon-anticodon base-pair geometry in these positions^42^. In particular, A1492 and A1493 adenosines form locally a triplex structure with the minor-groove of the codon-anticodon mini-helix (A-minor motif), see Fig. 8, panel B. Moreover, the conserved G530 base stabilizes the cognate codon-anticodon minihelix, promoting the latching of the decoding center. The interactions of G530 with the decoding center are provided by a hydrogen-bond network in the minor groove of the codon-anticodon helix. G530 not only contacts the riboses of the anticodon at the first and second base pairs, but also the opposed A1492 base, fastening the codon-anticodon minihelix in the decoding center^43^. These interactions appear to control domain closure of the 30S subunit^44^, accelerating the forward steps in decoding, thus influencing the dynamics of translation elongation (recently reviewed in^45^).

The evidence of minor-groove readout of the codon-anticodon mini-helix by the A1492, A1493 and G530 bears interesting implications: because of nucleoside biochemistry, weak (W) base-pairs (either A–U or U–A) have the same H-bond acceptor/donor profile in the minor groove. A-U or U-A are indistinguishable one from another with respect to the formation of an A-minor motif. The same applies for strong (S) base-pairs: C–G or G–C display a different profile of H-bond donor/acceptor with respect to weak base pairs, but are indistinguishable one from another in the minor groove^46^. Thus, out of the four different possible base pairs of two RNA nucleosides, only two possible hydrogen-bonding signatures can be discriminated in the minor groove, either weak (W) or strong (S) Fig. 8, panel C. Hence, if the A-minor motif and G530 form a structure able to monitor the correct base-pairing of the first two bases of the codon, through readout of the minor groove, then the dichotomic combination of S/W base pairs in these positions may impose different conformational arrangements of the 16S rRNA through A1492-A1493 dinucleotide interaction influencing the speed/rates of mRNA decoding by the ribosome. In addition, the latching of G530 may not be neutral to the H-bond acceptor or donor signature at the second position. In fact, the hydrogen bond network responsible for 30S domain closure reported in the case of the W character of the second base^43^ may be perturbed by the presence of the electron donor group in the case of S–S complementarity (see Fig. 8, panel D).

Strikingly, our analysis of circular code properties appears to point at a link between protein synthesis levels and the S/W dichotomy in the first and especially in the second bases of the codon. In particular, the results indicate a codon ordering where SWN codons confer the highest expression levels. In this respect, the theory of circular codes allowed to uncover the possible role played by the S/W dichotomy in the decoding process, and more in particular in the fascinating hypothesis that translation dynamics may be influenced by the electron-acceptor/donor signature of the second codon base. It is also worth noticing that without the lens of circular codes this property would have otherwise escaped from the analysis of synonymous sequence libraries, since the latter tend to vary mostly in the third (wobbling) position of the codon, and only marginally in the first two positions (only for degeneracy-6 codons). In conclusion, our results go beyond the simple view of the evolution of the genetic code to provide robustness against frame shifting errors. The existence of circular code motifs has been reported in many organisms^23^, and a formal conceptualization behind circular codes in biology has been brought about recently^30^. However, these aspects pertained mostly to the theoretical side of biology, and their impact and mechanistic explanations have been quite speculative to date. On the contrary, the present results allow, for the first time, to link circular code theory with translation rates and with the molecular events that take place in the ribosome decoding center.

For these reasons the theory of circular codes can be also seen as a promising tool for codon optimization of protein coding sequences to be used in biotechnological applications and for building sequence indicators for bioinformatics applications. If circular code properties play a role in translation then it will be possible to design dedicated experiments to verify their impact on expression rates and/or reading frame maintenance paving the way to a better understanding of the molecular mechanisms behind decoding.

**Table 2:** Codons of circular codes $X_{173}$ and $X_{192}$ together with their codon influence as in^5^ their codon usage in E.coli and the mRNA groove described as the Strong/Weak nature of the first two nucleotides of the codon. The columns are ordered in descending order according to the codon influence index for code $X_{173}$ (second column).

$X_{173}$				$X_{192}$			
*x*	Influence	Usage	Groove	YR$(\overleftarrow{{x}})$	Influence	Usage	Groove
GAT	23.85	3.22	SW	CGA	− 8.46	0.35	SS
GAA	22.51	3.97	SW	GGA	10.66	0.79	SS
GAC	16.19	1.91	SW	TGA	*	*	WS
GAG	15.51	1.80	SW	AGA	4.76	0.20	WS
GTA	11.32	1.09	SW	GCA	1.45	2.01	SS
GGC	5.05	2.98	SS	TAA	*	*	WW
GTT	4.92	1.82	SW	CCA	− 4.98	0.84	SS
GTG	3.80	2.63	SW	ACA	3.63	0.69	WS
CTC	3.53	1.11	SW	TCT	− 6.24	0.84	WS
CAC	2.31	0.98	SW	TGT	− 12.47	0.51	WS
CAG	1.75	2.90	SW	AGT	2.12	0.87	WS
AAC	1.53	2.16	WW	TGG	− 7.49	1.52	WS
CTG	0.99	5.31	SW	ACT	− 0.63	0.88	WS
GCC	0.97	2.57	SS	TTA	− 5.24	1.38	WW
GTC	0.31	1.53	SW	TCA	8.18	0.70	WS
ATT	− 0.19	3.04	WW	CCG	6.55	2.34	SS
TTC	− 3.95	1.65	WW	TCC	− 3.25	0.86	WS
AAT	− 5.25	1.76	WW	CGG	− 13.00	0.54	SS
TAC	− 5.45	1.22	WW	TGC	− 10.70	0.64	WS
ATC	− 6.71	2.52	WW	TCG	− 9.67	0.89	WS

## Methods: circular codes and comma free codes

In analogy with the transmission of a digital message, an efficient protein synthesis needs appropriate means to achieve the following fundamental tasks: (*i*) determine the points where translation should start and stop, (*ii*) avoid reading errors due to frame shifts, that is, ensure that the ribosome stays synchronized with the correct reading frame. The latter ability is called *reading frame maintenance* and is crucial since an error would result in a completely wrong protein. While the problem of punctuation signs has been elucidated to a great extent, the determinants of frame maintenance are still largely unknown. As mentioned in the Introduction, reading frame synchronization in mRNA translation was first studied in^25^, which proposed an elegant solution based on comma free codes. A **comma free** code is a special set of codons that allows to retrieve the normal reading frame anywhere in the sequence, provided this is composed only of codons of such code. The idea can be explained by means of the following simple example:

### *Example 1*

The comma free code *X* has two codons

1. Build a sequence with the codons of *X* (in green), for instance
2. Read it in the 3 possible frames:
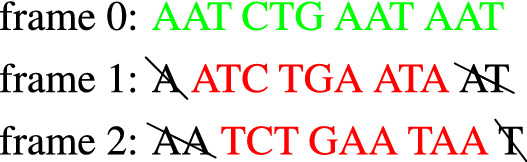
3. There is only one frame (frame 0) where all the codons belong to *X*: the *correct reading frame*. **None** of the codons (in red) read in frames + 1 and + 2 belong to *X*.

This holds for any sequence of arbitrary length formed with codons of *X*. In other words, if we form sequences by using codons of a comma free code and we read them with a frame shift then we end up immediately on a *forbidden* codon, i.e. a codon that does not belong to the code. Despite their appeal, comma free codes were proven not adequate and left aside, especially after the experiment of^47^, which showed that the codon TTT codes for the amino acid Phenylalanine but, for theoretical reasons, TTT cannot be a part of any comma-free code. In general, one can argue that it is not possible to identify good codons and build a code with them since all the 64 codons are used in protein synthesis; there are no forbidden or bad codons.

After 40 years from Crick’s proposal of comma-free codes, in^22^ it was found empirically that a weaker version of comma-free codes can be used to retrieve the normal reading frame. These are called *circular codes* and can be explained through the following example:

### *Example 2*

Assume that the circular code *X* has 3 codons

1. Form an arbitrary sequence with the codons of *X*, for instance:
2. Put it in a circle and read it in the 3 possible frames (the starting trinucleotide is coloured in blue):
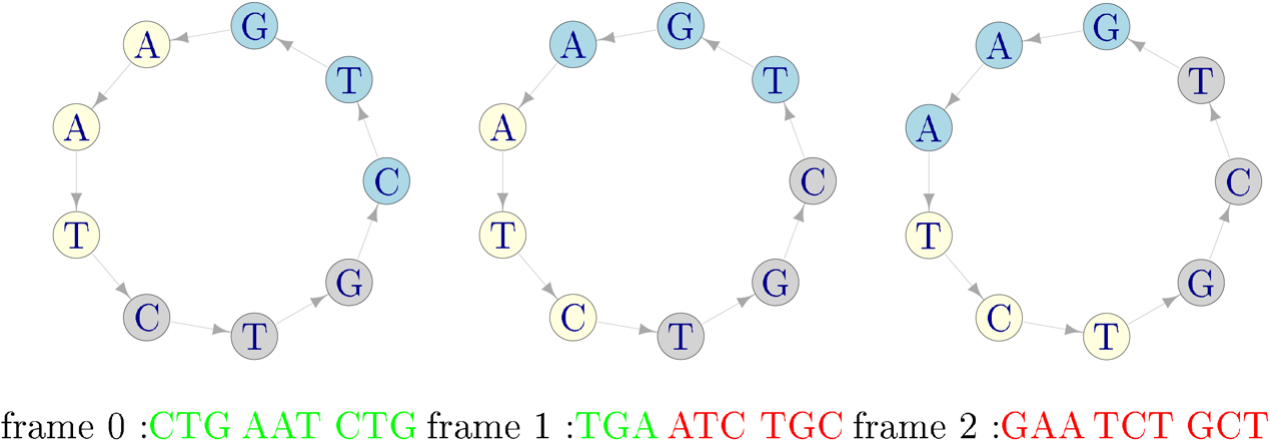
3. There is only one frame (frame 0) where all the codons belong to *X*: the *correct reading frame*, even if some of the codons read in frames +1 and +2 can belong to *X*.

As before, this holds for any sequence of arbitrary length formed with codons of *X*. The two examples pinpoint the difference between comma-free and circular codes: for comma-free codes a frame shift in the sequence invariably leads to a forbidden codon, whereas for circular codes this is not necessarily so and valid codons can be found when the sequence is read out of frame.

It is easy to show that a circular code can have at most 20 codons. If this is the case, the code is said *maximal*. The codes found in^22^ are maximal and have two additional properties: (1) they are *self complementary*: if a codon belongs to a code, then also its reverse complement belongs to the code; (2) they are $C^3$: the circular permutations of the codons of a circular code also form a circular code. We denote with $\alpha _1(x)$ and $\alpha _2(x)$ the two circular permutations of a codon *x*. For example, if $x= \mathrm {CTG}$ then $\alpha _1(x)= \mathrm {TGC}$ and $\alpha _2(x)= \mathrm {GCT}$, moreover, $\alpha _0(x):= x$. Note that the set of circular permutations ${\mathcal {A}}_3:= \{\alpha _0,\alpha _1,\alpha _2\}$, together with the usual composition operation, forms a group. There are exactly 216 circular codes that possess the three aforementioned properties i.e. they are maximal, self-complementary and $C^3$. In^30^ it has been proved that these 216 codes have special symmetry properties related to the transformations of the nucleotides. A transformation is a rule that maps the set of 4 nucleotides onto one of its 24 possible permutations. For instance, the transformation (AGT)(C) maps A to G, G to T, T to A and C to C, that is $\mathrm {A} \mapsto \mathrm {G}; \quad \mathrm {G} \mapsto \mathrm {T}; \quad \mathrm {T} \mapsto \mathrm {A}; \quad \mathrm {C} \mapsto \mathrm {C} \,.$ There are 8 special transformations that are related to the dihedral group of symmetry, that is, they represent the 8 symmetries of a square. These are shown in Table 3. Note that a single letter within brackets means that it is not transformed and for ease of notation it can be omitted. Hence, in the example above, (AGT)(C) becomes (AGT). The first four transformations of the list form a further group of symmetry (the Klein V group) that contains the identity plus three chemical transformations of the nucleotides^48^.

**Table 3:** Set of the 8 transformations of the nucleotides forming the dihedral group $D_8$. The first 4 transformations of the nucleotides form also the Klein V symmetry group. These are indicated as transformations in the rightmost column.

1.	(A)(T)(C)(G)	: A $\mapsto$ A; T $\mapsto$ T; C $\mapsto$ C; G $\mapsto$ G	Identity	(I)
2.	(AT)(CG)	: A $\mapsto$ T; T $\mapsto$ A; C $\mapsto$ G; G $\mapsto$ C	Strong/Weak	(SW)
3.	(AG)(CT)	: A $\mapsto$ G; G $\mapsto$ A; C $\mapsto$ T; T $\mapsto$ C	Purine/Pyrimidine	(YR)
4.	(AC)(GT)	: A $\mapsto$ C; C $\mapsto$ A; G $\mapsto$ T; T $\mapsto$ G	Keto/Amino	(KM)
5.	(A)(T)(CG)	: A $\mapsto$ A; T $\mapsto$ T; C $\mapsto$ G; G $\mapsto$ C		
6.	(AT)(C)(G)	: A $\mapsto$ T; T $\mapsto$ A; C $\mapsto$ C; G $\mapsto$ G		
7.	(ACTG)	: A $\mapsto$ C; C $\mapsto$ T; T $\mapsto$ G; G $\mapsto$ A		
8.	(AGTC)	: A $\mapsto$ G; G $\mapsto$ T; T $\mapsto$ C; C $\mapsto$ A		

An important result proved in^30^ states that by means of the 8 above transformations it is possible to partition the 216 codes in 27 equivalence classes, see Table 4. Each equivalence class contains 8 circular codes related through the 8 transformations of the dihedral group shown in Table 3, namely: $D_8 = \{\mathrm {I},(\mathrm {AT}),(\mathrm {CG}),{{\,\mathrm{SW}\,}},{{\,\mathrm{YR}\,}},(\mathrm {AGUC}),(\mathrm {ACUG}),{{\,\mathrm{KM}\,}}\}.$ Note that, strictly speaking, the above group of transformations does not coincide with $D_8$ but is isomorphic to it. Formally, two circular codes $X_i$ and $X_j$ are equivalent iff there exists a transformation $\pi \in D_8$ such that $X_i = \pi (X_j)$. The classification in equivalence classes is one of the key aspects connecting the theory of circular codes with the experimental results on protein expression levels. In Table 4 we show an example of one of the 27 equivalence classes. For instance, from the first row of the table we can see that codon AAC belongs to code number 173 ($X_{173}$) whereas its Keto-Amino transformation KM(AAC) = CCA belongs to code 192 ($X_{192}$) and so on. We adopt the following notation to combine the action over the code *X* of the transformations $\pi \in D_8$ with circular permutations: $X^{f,\pi } = \pi (\alpha _f(X)) = \alpha _f(\pi (X))$, where $f=0,+1,+2$.

**Table 4:** Equivalence class formed by eight circular codes. Each column represents one of the 216 circular codes $X_i$, where $i \in \{1,\dots ,216\}$. The codes are related through the group of transformations $D_8$. For instance AAC $\in X_{173}$ and KM(AAC) = CCA $\in X_{192}$.

	$\mathrm {I}$	${(\mathrm {AT})}$	${(\mathrm {CG})}$	$\mathrm {SW}$	$\mathrm {YR}$	${(\mathrm {ACTG})}$	${(\mathrm {AGTC})}$	$\mathrm {KM}$
	$X_{173}$	$X_{176}$	$X_{203}$	$X_{206}$	$X_{183}$	$X_{182}$	$X_{193}$	$X_{192}$
1	AAC	TTC	AAG	TTG	GGT	GGA	CCT	CCA
2	GTT	GAA	CTT	CAA	ACC	TCC	AGG	TGG
3	AAT	TTA	AAT	TTA	GGC	GGC	CCG	CCG
4	ATT	TAA	ATT	TAA	GCC	GCC	CGG	CGG
5	ATC	TAC	ATG	TAG	GCT	GCA	CGT	CGA
6	GAT	GTA	CAT	CTA	AGC	TGC	ACG	TCG
7	CAC	CTC	GAG	GTG	TGT	AGA	TCT	ACA
8	GTG	GAG	CTC	CAC	ACA	TCT	AGA	TGT
9	CAG	CTG	GAC	GTC	TGA	AGT	TCA	ACT
10	CTG	CAG	GTC	GAC	TCA	ACT	TGA	AGT
11	CTC	CAC	GTG	GAG	TCT	ACA	TGT	AGA
12	GAG	GTG	CAC	CTC	AGA	TGT	ACA	TCT
13	GAA	GTT	CAA	CTT	AGG	TGG	ACC	TCC
14	TTC	AAC	TTG	AAG	CCT	CCA	GGT	GGA
15	GAC	GTC	CAG	CTG	AGT	TGA	ACT	TCA
16	GTC	GAC	CTG	CAG	ACT	TCA	AGT	TGA
17	GCC	GCC	CGG	CGG	ATT	TAA	ATT	TAA
18	GGC	GGC	CCG	CCG	AAT	TTA	AAT	TTA
19	GTA	GAT	CTA	CAT	ACG	TCG	AGC	TGC
20	TAC	ATC	TAG	ATG	CGT	CGA	GCT	GCA

### Coverage of a circular code

The **coverage** of a circular code over a specific sequence or organism, is a key quantity to study the role played by circular codes in translation. It is the cumulative codon usage of the codons belonging to a code and can be seen as a measure of the “goodness” of a code, see also^29^. It can be interpreted as a sort of aggregate codon usage of the set of codons of the code. In the following we provide a rigorous mathematical definition.

Given a genome *i*, we define its codon distribution (or codon usage) ${\mathbf {p}}_i$ over the set of codons of ${\mathcal {B}}^3$ as:

**Table:** 

Codons	$x_{1}$	$\dots$	$x_{k}$	$\dots$	$x_{64}$
Usage	$p_{1i}$	$\dots$	$p_{ki}$	$\dots$	$p_{64\,i}$

where $x_k \in {\mathcal {B}}^3$ and $p_{ki} \in {\mathbf {p}}_i$. Next, we define the coverage of a code as the cumulative codon usage over the set of codons that compose the code.

#### **Definition 1**

Given a circular code $X_j \in {\mathfrak {C}}$ where $j = 1,\dots ,216$ and a genome *i*, we define as $\mathrm {C}_{ij}$ the coverage of code $X_j$ over genome *i*: $\mathrm {C}_{ij} = \sum _{k=1}^{64} p_{ki} \; I_{X_j}(x_k),$ where $I_A(x)$ is the indicator function, i.e. $I_A(x) = 1$ if $x \in A$, 0 otherwise.

Clearly, the coverage ranges in [0, 1].

#### *Example 3*

Consider the sequence $\hbox {CAT}\; \hbox {CTG}\; \hbox {AAT}\; \hbox {GGA}\;$ CTG and the two codes $X_1 = \{\mathrm {CTG}, \mathrm {AAT}\}$, $X_2 = \{\mathrm {GGA}, \mathrm {TGT}\}$. The codon usage of the sequence is

**Table:** 

Codons	CAT	CTG	AAT	GGA
Usage	1/5	2/5	1/5	1/5

The coverage of $X_1$ results $2/5 + 1/5 = 3/5 = 0.60$, and that of $X_2$ results $1/5 = 0.20$.

Note that the coverage, as defined in^29^, accounts for the simultaneous coverage of a code in the three frames but here we consider the coverage in the three frames separately. Also, other indicators have been proposed to study the properties of circular codes, see e.g.^24^ and references therein, but the coverage appears the most appropriate to our aim.

## Supplementary information

Supplementary material 1 (pdf 1385 KB)
