# Supplementary material for: A role for circular code properties in translation
Source: Sci Rep. 2021 Apr 28;11:9218. doi: 10.1038/s41598-021-87534-y (PMC8080828; doi:10.1038/s41598-021-87534-y)
Supplement: Supplementary file 1 — Supplementary material 1 (pdf 1385 KB) [file 41598_2021_87534_MOESM1_ESM.pdf]

Supplement to:
A role for circular code properties in translation

Simone Giannerini[1,*], Diego Luis Gonzalez[1,3], Greta Goracci[1], and Alberto Danielli[2]

[*]Corresponding author: simone.giannerini@unibo.it
[1]Department of Statistical Sciences, University of Bologna, Bologna, 40126, Italy.
[2]Department of Pharmacy and Biotechnology, University of Bologna, Bologna, 40126, Italy.
[3]Institute for Microelectronics and Microsystems - Bologna Unit, CNR, Bologna, 40129, Italy.

ABSTRACT

This supplement follows the same sectioning of the article and contains additional results for each section.

2 Results

2.1 Universal properties of Circular codes' coverage

We have analyzed the whole Codon Usage Database available at http://www.kazusa.or.jp/codon/[1]. It contains 35799 organisms and 3,027,973 complete protein coding genes (CDS). After some cleaning and removing the mitochondrial genomes we end up with 25528 nuclear genomes. In Table 1 we report a brief summary of the database. In Table 2 of the main article we show the coverage (in percentage), the rank over the 216 codes and the rank within a class for the equivalence class composed of the 8 codes shown in Table 4 of the main article. The ranks inside the equivalence class show a universal ordering among the 8 codes, irrespective of the species-specific codon usage. In particular, the worst code within each class (code with the least coverage) invariably coincides with the chemical Keto-Amino transformation of the best one. As we will show, this property holds for all the equivalence classes. Here and in the following we focus on the 16 equivalence classes for which the best and the worst codes (w.r.t coverage) are disjoint sets.

The results shown in Table 2 demonstrate that universal symmetry properties of coding sequences emerge when analyzed through the theoretical framework of circular codes, irrespectively of the species-specific codon-usage. Moreover, within each equivalence class, the Keto-Amino transformation of the code possessing the best coverage always leads to the worst covering code of the same class. This establishes an important connection between the codon usage, the Keto-Amino (KM) and the Purine-Pyrimidine (YR) chemical transformation. Behind the heterogeneity of the codon usage, there is a universal ordering structure conserved across domains of life, grounded by a theoretical framework of circular codes. The aforementioned properties emerge only if we consider these special set of codons (codes) and does not necessarily hold at the level of single codons. This is substantiated further by means of a statistical test.

2.1.1 A bootstrap test

In this section we present a bootstrap test to explore the relation between the coverages of the best and the worst code of an equivalence class. In the previous section we have shown that, within each equivalence class, the code $X \in \mathfrak{C}$ that has the best coverage is (almost) unique. Also, the code that has the worst coverage is $\mathrm{KM}(X)$, the Keto-Amino (Rumer) transformation of code X. Is this result due to sheer chance?

species	# organisms
bacteria	4918
animals	6921
viral	1956
plants	11733
total	25528

domain	# organisms
prokaryotes	4918
eukaryotes	15598
total	20516

Table 1. Description of the codon usage database analyzed. The right table shows the aggregation by domain, excluding viruses and phage.

coverage

best	X_{173}	X_{23}	X_{98}	X_{166}	X_{4}	X_{172}	X_{21}	X_{24}	X_{97}	X_{171}	X_{3}	X_{165}	X_{115}	X_{161}	X_{41}	X_{107}
bacteria	46.4	46.5	46.0	44.3	44.4	43.5	43.7	42.9	43.2	43.0	41.6	41.4	40.8	40.9	39.0	38.6
animals	42.0	41.3	41.6	41.3	40.6	40.0	39.3	40.1	39.6	39.4	38.6	39.3	39.4	38.7	37.9	36.4
viral	43.2	42.7	42.0	42.6	42.1	41.0	40.5	41.1	39.8	40.6	39.9	40.4	40.5	40.0	38.7	37.5
plants	39.7	40.1	39.6	39.6	40.0	40.0	40.5	40.4	39.9	40.8	40.3	39.9	40.3	40.7	38.8	38.6
worst	X_{192}	X_{87}	X_{53}	X_{191}	X_{86}	X_{195}	X_{91}	X_{57}	X_{54}	X_{208}	X_{90}	X_{194}	X_{136}	X_{207}	X_{93}	X_{146}
bacteria	18.1	19.7	19.9	17.1	18.7	17.0	18.6	20.5	18.8	22.1	17.6	16.0	19.5	21.1	22.7	23.5
animals	23.4	23.8	23.4	22.4	22.8	21.7	22.1	24.2	21.7	24.6	21.1	20.7	23.2	23.6	24.3	25.7
viral	22.4	22.5	22.8	21.6	21.7	20.4	20.5	23.5	20.9	23.6	19.7	19.6	22.7	22.8	23.7	25.0
plants	23.5	25.2	23.9	22.9	24.7	21.7	23.5	22.4	22.2	24.2	22.9	21.2	21.9	23.6	23.7	25.7

absolute rank

best	X_{173}	X_{23}	X_{98}	X_{166}	X_{4}	X_{172}	X_{21}	X_{24}	X_{97}	X_{171}	X_{3}	X_{165}	X_{115}	X_{161}	X_{41}	X_{107}
bacteria	2	1	3	7	6	16	13	19	17	18	22	24	29	28	35	39
animals	2	7	3	6	9	12	18	11	14	16	21	17	15	20	27	37
viral	2	4	9	6	8	12	15	11	22	14	20	17	16	19	29	35
plants	16	9	18	17	12	11	4	5	15	1	7	14	8	2	20	22
worst	X_{192}	X_{87}	X_{53}	X_{191}	X_{86}	X_{195}	X_{91}	X_{57}	X_{54}	X_{208}	X_{90}	X_{194}	X_{136}	X_{207}	X_{93}	X_{146}
bacteria	212	207	206	214	210	215	211	205	209	196	213	216	208	201	191	183
animals	208	205	207	211	210	214	212	202	213	200	215	216	209	206	201	189
viral	209	208	203	211	210	214	213	202	212	201	215	216	207	205	200	185
plants	208	195	202	210	198	215	209	212	213	201	211	216	214	205	203	187

relative rank

best	X_{173}	X_{23}	X_{98}	X_{166}	X_{4}	X_{172}	X_{21}	X_{24}	X_{97}	X_{171}	X_{3}	X_{165}	X_{115}	X_{161}	X_{41}	X_{107}
bacteria	1	1	1	1	1	1	1	1	1	1	1	1	1	1	1	1
animals	1	1	1	1	1	1	1	1	1	1	1	1	1	1	1	1
viral	1	1	1	1	1	1	1	1	1	1	1	1	1	1	1	1
plants	1	1	1	1	1	1	1	1	1	1	1	1	1	1	1	1
worst	X_{192}	X_{87}	X_{53}	X_{191}	X_{86}	X_{195}	X_{91}	X_{57}	X_{54}	X_{208}	X_{90}	X_{194}	X_{136}	X_{207}	X_{93}	X_{146}
bacteria	8	8	8	8	8	8	8	8	8	8	8	8	8	8	8	8
animals	8	8	8	8	8	8	8	8	8	8	8	8	8	8	8	8
viral	8	8	8	8	8	8	8	8	8	8	8	8	8	8	8	7
plants	8	8	8	8	8	8	8	8	8	8	8	8	8	8	8	8

Table 2. Coverage (upper panel), absolute ranks (mid panel) and relative ranks (lower panel) for the best and worst codes of the 16 equivalence classes highlighted in bold in Table 11 (the best and the worst codes are disjoint sets). The universality of the results is clear if we consider the ranks within classes: for instance the coverage of code X_{173} (X_{192}) for bacteria is 46.4 (18.1) (upper panel). It is not the highest (lowest) among the 216 codes, indeed it ranks 2nd (212th) (mid panel). However, it is always the highest (lowest) within its class (lower panel).

.

It is natural to expect that the more a set of codons is recurrent then the less recurrent are codons that do not belong to that set. We have selected from the database the genomes with at least 1 million codons. There are 291 such genomes on which we have computed the coverage of the pairs of codes X_{173}, X_{192} and X_{23}, X_{87}. These are shown in Figure 1, where we have superimposed the following quadratic least square fits (blue points):

$$C_{i,192} = \beta_{0,173} + \beta_{1,173} C_{i,173} + \beta_{2,173} C_{i,173}^2 + \varepsilon_i$$
$$C_{i,87} = \beta_{0,23} + \beta_{1,23} C_{i,23} + \beta_{2,23} C_{i,23}^2 + \varepsilon_i$$

where $i = 1, \ldots, 291$ genomes. Clearly, the quadratic fit accounts for 87% of the observed variability. Hence, the question is: is this negative correlation due to the fact that the more one set is recurrent, then the less recurrent is its complement? In other words, is this correlation compatible with the natural correlation produced by a random choice of codons? For instance, in the equivalence class of Table 4 the best and the worst codes are X_{173} and X_{192}, respectively. Now, we show that $C_{i,192}$, i.e. the coverage of X_{192} is significantly smaller than the coverage of a random set of 20 codons taken from those who do not belong to the best code X_{173}. Formally, denote with C_{ij} and $\mathrm{C}_{ij'}$ the coverage of the codes X_j and $X_{j'} = \mathrm{KM}(X_j)$ over a genome i. Also, let $D = \mathscr{B}^3 \setminus X_j$ be the set of 44 codons that do not belong to X_j. In statistical terms the system of hypotheses results

$$\begin{cases} H_0 : \mathrm{C}_{ij} \text{ is compatible with } \bar{\mathrm{C}}_{ij} \\ H_1 : \mathrm{C}_{ij} \text{ is not compatible with } \bar{\mathrm{C}}_{ij} \end{cases} \tag{1}$$

where $\bar{\mathrm{C}}_{ij}$ is the following random variable: coverage on genome i of a random set of 20 codons taken from D. In order to test this hypothesis we implement the following bootstrap scheme:

1. Compute C_{ij} and $\mathrm{C}_{ij'}$ the coverage of the codes X_j and $X_{j'} = \mathrm{KM}(X_j)$ over a genome i.

2. Resample without replacement B sets of 20 elements from $D = \mathscr{B}^3 \setminus X_j$ and compute the coverage over genome i for each resample $\bar{X}^*_{jb}$, with $b = 1 \ldots, B$:

Set	$\bar{X}^*_{j1}$	$\ldots$	$\bar{X}^*_{jB}$
Coverage	$\bar{\mathrm{C}}^*_{ij1}$	$\ldots$	$\bar{\mathrm{C}}^*_{ijB}$

   The set of resampled codes can be made homogeneous with respect to the GC content by imposing that their GC content be equal to that of the original code $X_{j'}$.

3. Compare the coverage $\mathrm{C}_{ij'}$ with the quantiles of the empirical distribution function of the coverage $\bar{F}^*_{ij}$. Alternatively, compute the bootstrap p-value $p = B^{-1} \sum_{b=1}^{B} I(\bar{\mathrm{C}}^*_{ijb} > \mathrm{C}_{ij'})$.

At Step 2 of the algorithm the resampling of the sets of codons from D can be performed in two ways: i) using a uniform distribution over D; ii) using the codon distribution of genome i over D. The hypotheses can have a different biological meaning/interpretation. In brief, the first hypothesis assumes that all the 216 codes are equally likely to occur in practice and exist independently from the codon usage of the genomes. The second hypothesis, instead, assumes that the occurrence of a circular codes is related to the codon usage of a given genome. Since the results point to a universal relationship which is independent of the codon usage in genomes we tend to support the first hypothesis. In any case, we have performed the tests for both hypotheses and we show the results in Figure 1. The red lines correspond to the Monte Carlo rejection bands for hypothesis i): the occurrence of the codes is uniform; the green lines are the rejection bands for hypothesis ii): the occurrence of the codes depends on the codon usage. In both cases $\alpha = 0.001$ (i.e. $B = 9999$ bootstrap replicates). The results show clearly that in both scenarios H_0 is rejected so that the negative correlation observed cannot be ascribed to random fluctuations and goes well beyond the naturally induced correlation.

2.2 Universal frame marks in coding sequences.

In this section we report the extended analysis in the three reading frames over the whole set of complete coding sequences for 25 organisms. The description of the genomes is reported in Table 3.

We present the coverage ranks within 16 equivalence classes of the set of maximal, self-complementary and C^3 circular codes. Within such classes, the best and the worst codes are disjoint sets. In particular, Tables 4 and 5 show the coverage (relative) ranks in frame for the best and worst codes, respectively. Similarly, Tables 6 and 7 show the same analysis where the coding sequences are in the reading frame +1 and the set of codes are obtained from the first circular permutation $\alpha_1(\cdot)$ of the

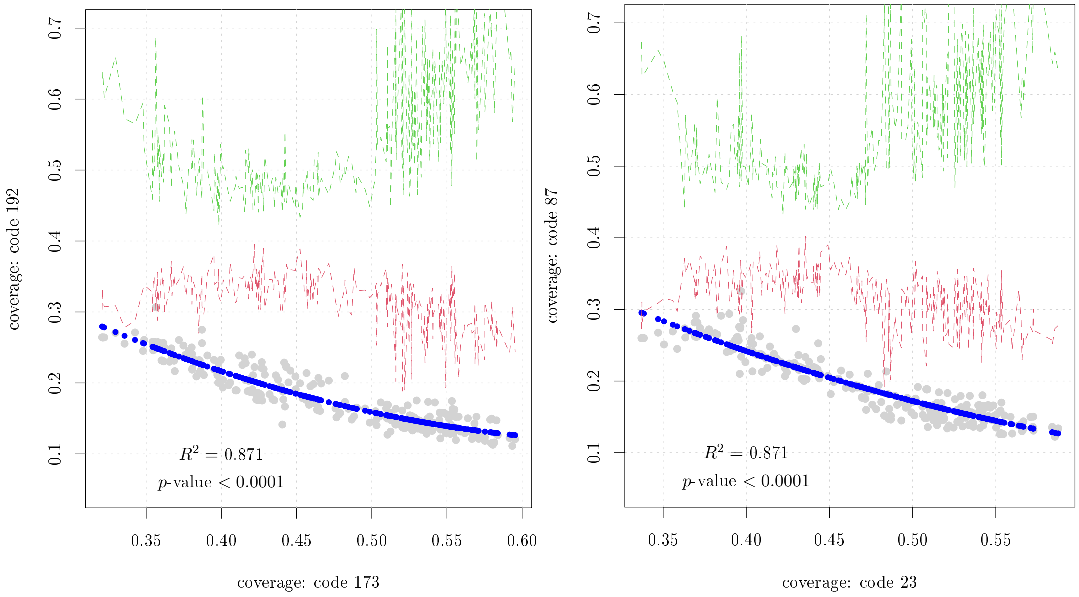

Figure 1. Coverage for the best and worst codes of two equivalence classes, computed over 291 genomes with more than 1 million codons (gray points). The blue points represent a quadratic fit and the associated R^2 and p-value are reported under it. The green and red lines correspond to the bootstrap rejection bands under the null hypothesis at $\alpha = 0.001$ that the observed relation is produced by chance, as follows: red line, hypothesis 1: the occurrence of the codes is uniform; green line, hypothesis 2: the occurrence of the codes follows the codon usage. In both cases the null hypothesis that the negative correlation is due to chance is clearly rejected.

usual set of 216 codes used in frame. Finally, Tables 8 and 9 present the same analysis for frame +2 sequences and the second circular permutation $\alpha_2(\cdot)$ of the set of codes. In order to show that this universal property observed in all the reading frames is connected to the circularity property of the codes, we have generated 1000 random codes with the following properties: *i*) self complementarity, *ii*) maximality, *iii*) they do not contain stop codons or periodic codons (AAA, TTT, CCC, GGG), *iv*) they are **not** circular. Then, for each random code X we have derived its equivalence class by applying the 8 transformations in D_8. Also, we have generated the circularly permuted versions of the codes in the equivalence class. Hence, a given random code X generates 24 (random) codes $X^{f,\pi}$, where $f = 0, +1, +2$ and $\pi \in D_8$, leading to 8000 random codes for each frame. First, we have identified a unique class representative among the 25 organisms by selecting the code that ranks first within its class for most organisms; we denote such code with $X^{0,I}$. In this way we have replicated the group theoretic framework for random codes that share the same properties as the 216 circular codes except circularity. We define the random code $X^{0,I}$ to behave like a circular code if it shows the same ranking properties observed in Table 4–9. In other words, we count the proportion of random codes satisfying the following condition:

$$\left\{(X^{f,I} \text{ ranks 1st or 2nd }) \wedge (X^{f,KM} \text{ ranks 7th or 8th}), \text{ for all organisms, and for each frame } f = 0, +1, +2\right\}.$$

We call such proportion p_r and compare it with the proportion observed among the circular codes (p_c) as follows:

$$H_0 : p_c - p_r = 0 \text{ versus } H_1 : p_c - p_r > 0,$$

Under H_0, the large sample distribution of the test statistic is Gaussian

$$\hat{p}_c - \hat{p}_r \sim N\left(0, \frac{p_c(1-p_c)}{27} + \frac{p_r(1-p_r)}{1000}\right) \qquad Z = \frac{\hat{p}_c - \hat{p}_r}{\sqrt{\frac{\hat{p}_c(1-\hat{p}_c)}{27} + \frac{\hat{p}_r(1-\hat{p}_r)}{1000}}} \sim N(0,1),$$

where $\hat{p}_c$ and $\hat{p}_r$ are the estimators for p_c and p_r. The value of the test statistic results 5.37 with a corresponding p-value of 3.9×10^{-8}. The results indicate that the property of circularity is strictly connected to the universal ranking structure of the coverage that has been observed in coding sequences.

2.3 The universal properties of circular codes are absent in introns.

The structure uncovered in coding sequences is completely absent in introns as it is shown in Table 10, where we present the mean coverage over 225 intron sequences of A.thaliana. Clearly, there is no organization implied by circular codes within

organism	no. sequences	no. codons
AeropyrumPernix	713	228864
Arabidopsis.Thaliana	151245	66162308
Archaeoglobus	3757	1180343
Bacillus.subtilis	104992	34879326
Caenorhabditis.elegans	3347	2110402
DanioRerio	24118	10557602
Drosophila.melanogaster	12606	8945564
Escherichia.coli	3983	1346730
Helicobacter.pylori	2392	848550
Homo.Sapiens	140450	58477968
Leishmania.major	8239	5285329
M.Xanthus	5037	2043663
Methanosarcina	2963	1039833
MusMusculus	92857	41549270
Myxococcus	5037	2043663
OryzaSativa	65554	25953002
P.Horikoshii	1583	459156
Plasmodiumfalciparum3D7	5259	4121429
Pyrococcus	1441	462830
Schizosaccharomyces.Pombe	7711	3787977
Staphylococcus.aureus	1977	648703
Streptomyces.coelicolorA3	5202	1828585
Sulfolobus.solfataricus	9674	2969225
Thermoplasma.acidophilum	1150	379203
ZeaMays	70650	25168719

Table 3. Description of the organisms whose complete CDS set has been analyzed in the three reading frames.

introns. These results are consistent with the findings in the literature where it is shown that circular code properties are either absent or diminished in non-coding regions of the genome, see e.g.[2] and references therein.

**coverage**	X_{173}	X_{176}	X_{203}	X_{206}	X_{183}	X_{182}	X_{193}	X_{192}
frame 0	29.5	28.9	30.3	29.7	29.4	29.3	29.6	29.6
frame +1	29.1	29.5	28.8	29.2	29.2	29.3	28.9	29.0
frame +2	28.9	29.3	28.9	29.3	29.0	29.1	29.2	29.2
**relative rank**	X_{173}	X_{176}	X_{203}	X_{206}	X_{183}	X_{182}	X_{193}	X_{192}
frame 0	4	1	8	7	3	2	5	5
frame +1	4	8	1	5	5	7	2	3
frame +2	1	7	1	7	3	4	5	5

Table 10. Mean coverage (upper panel) and relative rank (lower panel) of the 8 circular codes forming the equivalence class presented in Table 4 computed over 225 intron sequences of A. thaliana.

In conclusion, each circular code has a distinct degree of coverage with respect to the species-specific codon usage of distinct organisms, according also to the GC content. Notably, however, there are recurring properties, linking the coverage inside equivalence classes with the set of transformations of the codons of the codes.

2.4 Circular codes and *in vivo* translation speed

We have tested the significance of the result by comparing the correlation coefficient obtained on the circular codes $\hat{\rho}_c = 0.835$ with that obtained on the random codes $\hat{\rho}_r = 0.573$. The system of hypotheses is:

$$H_0 : \rho_c = \rho_r \text{ versus } H_1 : \rho_c > \rho_r.$$

	X_{173}	X_{23}	X_{98}	X_{166}	X_4	X_{172}	X_{21}	X_{24}	X_{97}	X_{171}	X_3	X_{165}	X_{115}	X_{161}	X_{41}	X_{107}
AeropyrumPernix	1	1	2	1	1	1	1	1	2	1	1	1	1	1	1	1
Arabidopsis.Thaliana	1	1	1	1	1	1	1	1	1	1	1	1	1	1	1	1
Archaeoglobus	1	1	2	1	1	1	1	1	2	1	1	1	1	1	1	1
Bacillus.subtilis	1	1	1	1	1	1	1	1	1	1	1	1	1	1	1	2
Caenorhabditis.elegans	1	1	1	1	1	1	1	1	1	1	1	1	1	1	2	1
DanioRerio	1	1	1	1	1	1	1	1	1	1	1	1	1	1	1	1
Drosophila.melanogaster	1	1	1	1	1	1	1	1	1	1	1	1	1	1	1	1
Escherichia.coli	1	1	1	1	1	1	1	1	1	1	1	1	2	1	1	1
Helicobacter.pylori	1	1	1	2	2	1	1	1	1	1	1	1	1	1	2	1
Homo.Sapiens	1	1	1	1	1	1	1	1	1	1	1	1	1	1	1	1
Leishmania.major	1	1	2	1	1	1	1	1	1	1	1	1	1	1	1	1
M.Xanthus	1	1	1	1	1	1	1	1	1	1	1	1	1	1	1	1
Methanosarcina	1	1	1	1	1	1	1	1	1	1	1	1	1	1	1	3
MusMusculus	1	1	1	1	1	1	1	1	1	1	1	1	1	1	1	1
Myxococcus	1	1	1	1	1	1	1	1	1	1	1	1	1	1	1	1
OryzaSativa	1	1	1	1	1	1	1	1	1	1	1	1	1	1	1	1
P.Horikoshii	1	1	2	1	1	1	1	1	2	1	1	1	1	1	1	1
Plasmodiumfalciparum3D7	1	1	1	1	1	1	1	1	1	1	2	2	2	2	1	2
Pyrococcus	1	1	2	1	1	1	1	1	2	1	1	1	1	1	1	1
Schizosaccharomyces.Pombe	1	1	1	1	1	1	1	1	1	1	1	1	1	1	1	1
Staphylococcus.aureus	1	1	1	3	1	1	1	1	1	1	2	3	2	2	1	2
Streptomyces.coelicolorA3	1	1	2	1	1	1	1	1	1	1	1	1	1	1	2	3
Sulfolobus.solfataricus	1	1	1	1	1	1	1	1	1	1	1	1	1	1	2	2
Thermoplasma.acidophilum	1	1	2	1	1	1	1	1	2	1	1	1	1	1	1	1
ZeaMays	1	1	2	1	1	1	1	1	1	1	1	1	1	1	1	1

Table 4. FRAME 0: relative coverage rank over 25 genomes for the 16 codes identified as best codes. No matter the organism, such codes are almost invariably ranked first within their equivalence class.

	X_{192}	X_{87}	X_{53}	X_{191}	X_{86}	X_{195}	X_{91}	X_{57}	X_{54}	X_{208}	X_{90}	X_{194}	X_{136}	X_{207}	X_{93}	X_{146}
AeropyrumPernix	8	8	8	8	8	8	8	8	8	8	8	8	8	8	8	8
Arabidopsis.Thaliana	8	8	7	8	8	8	8	8	8	8	8	8	8	8	8	8
Archaeoglobus	8	8	8	8	8	8	8	8	8	8	8	8	8	8	8	8
Bacillus.subtilis	8	8	7	8	8	8	8	8	8	8	8	8	8	8	8	8
Caenorhabditis.elegans	8	8	7	8	8	8	8	8	8	8	8	8	8	8	8	8
DanioRerio	8	8	8	8	8	8	8	8	8	8	8	8	8	8	8	7
Drosophila.melanogaster	8	8	7	8	8	8	8	8	8	8	8	8	8	8	8	8
Escherichia.coli	8	8	7	8	8	8	8	8	8	8	8	8	8	8	8	7
Helicobacter.pylori	8	8	8	8	8	8	8	8	8	8	8	8	8	8	7	7
Homo.Sapiens	8	8	8	8	8	8	8	8	8	8	8	8	8	8	8	8
Leishmania.major	8	8	8	8	8	8	8	8	8	8	8	8	8	8	8	8
M.Xanthus	8	8	8	8	8	8	8	8	8	8	8	8	8	8	8	8
Methanosarcina	8	8	7	8	8	8	8	8	8	8	8	8	8	8	8	8
MusMusculus	8	8	8	8	8	8	8	8	8	8	8	8	8	8	8	7
Myxococcus	8	8	8	8	8	8	8	8	8	8	8	8	8	8	8	8
OryzaSativa	8	8	8	8	8	8	8	8	8	8	8	8	8	8	8	8
P.Horikoshii	8	8	8	8	8	8	8	8	8	8	8	8	8	8	8	8
Plasmodiumfalciparum3D7	7	7	6	8	8	8	8	7	8	7	8	8	8	8	8	8
Pyrococcus	8	8	8	8	8	8	8	8	8	8	8	8	8	8	8	8
Schizosaccharomyces.Pombe	8	8	8	8	8	8	8	8	8	8	8	8	8	8	7	8
Staphylococcus.aureus	7	7	6	8	8	8	7	8	7	7	8	8	8	8	8	8
Streptomyces.coelicolorA3	8	8	8	8	8	8	8	8	8	8	8	8	8	8	8	8
Sulfolobus.solfataricus	8	8	8	8	8	8	8	8	8	8	8	8	8	8	8	8
Thermoplasma.acidophilum	8	8	8	8	8	8	8	8	8	8	8	8	8	8	8	8
ZeaMays	8	8	8	8	8	8	8	8	8	8	8	8	8	8	8	8

Table 5. FRAME 0: relative coverage rank over 25 genomes for the 16 codes identified as worst codes. No matter the organism, such codes are almost invariably ranked eighth within their equivalence class. They are obtained as the Keto-Amino transformation of the best codes.

$\alpha_1(\cdot)$	X_{173}	X_{23}	X_{98}	X_{166}	X_4	X_{172}	X_{21}	X_{24}	X_{97}	X_{171}	X_3	X_{165}	X_{115}	X_{161}	X_{41}	X_{107}
AeropyrumPernix	1	1	2	1	1	1	1	1	2	1	1	1	1	1	1	1
Arabidopsis.Thaliana	1	1	2	1	1	1	1	1	2	1	1	1	1	1	2	1
Archaeoglobus	1	1	3	1	1	1	1	1	2	1	1	1	1	1	2	2
Bacillus.subtilis	1	1	1	1	1	1	1	1	1	1	1	1	1	1	2	2
Caenorhabditis.elegans	1	1	2	1	1	1	1	1	2	1	1	1	1	1	1	1
DanioRerio	1	1	2	1	1	1	1	1	2	1	1	1	1	1	1	1
Drosophila.melanogaster	1	1	2	1	1	1	1	1	2	1	1	1	1	1	1	1
Escherichia.coli	1	1	1	1	1	1	1	1	1	1	1	1	1	1	2	1
Helicobacter.pylori	1	1	1	1	1	1	1	1	1	1	1	1	1	2	3	2
Homo.Sapiens	1	1	2	1	1	1	1	1	2	1	1	1	1	1	1	1
Leishmania.major	1	1	2	1	1	1	1	1	2	1	1	1	1	1	1	1
M.Xanthus	2	2	2	1	1	1	1	1	1	1	1	1	1	1	2	2
Methanosarcina	1	1	2	1	1	1	1	1	2	1	1	1	1	1	2	2
MusMusculus	1	1	2	1	1	1	1	1	2	1	1	1	1	1	1	1
Myxococcus	2	2	2	1	1	1	1	1	1	1	1	1	1	1	2	2
OryzaSativa	1	1	2	1	1	1	1	1	2	1	1	1	1	1	1	1
P.Horikoshii	1	1	2	1	1	1	1	1	2	1	1	1	1	1	1	2
Plasmodiumfalciparum3D7	1	1	1	1	1	1	1	1	1	1	2	2	1	1	1	1
Pyrococcus	1	1	2	1	1	1	1	1	2	1	1	1	1	1	1	2
Schizosaccharomyces.Pombe	1	1	2	1	1	1	2	1	1	2	1	1	1	1	2	1
Staphylococcus.aureus	1	1	1	1	1	1	1	1	1	1	1	1	1	1	2	2
Streptomyces.coelicolorA3	2	1	2	1	1	1	1	1	1	1	1	1	1	1	1	3
Sulfolobus.solfataricus	1	1	1	1	1	1	1	1	1	1	1	1	1	1	2	2
Thermoplasma.acidophilum	2	1	3	1	1	1	1	1	2	1	1	1	1	1	1	2
ZeaMays	1	1	2	1	1	1	1	1	2	1	1	1	1	1	1	1

Table 6. FRAME +1: relative coverage rank over 25 genomes for the 16 codes identified as best codes. No matter the organism, such codes are almost invariably ranked first within their equivalence class.

$\alpha_1(\cdot)$	X_{192}	X_{87}	X_{53}	X_{191}	X_{86}	X_{195}	X_{91}	X_{57}	X_{54}	X_{208}	X_{90}	X_{194}	X_{136}	X_{207}	X_{93}	X_{146}
AeropyrumPernix	8	8	8	8	8	8	8	8	8	8	8	8	8	8	8	8
Arabidopsis.Thaliana	8	8	8	8	8	8	8	8	8	8	8	8	8	8	8	7
Archaeoglobus	8	8	7	8	8	8	8	8	8	8	8	8	8	8	8	7
Bacillus.subtilis	8	8	8	8	8	8	8	8	8	8	8	8	8	8	8	7
Caenorhabditis.elegans	8	8	7	8	8	8	8	8	8	8	8	8	8	8	8	7
DanioRerio	8	8	6	8	8	8	8	8	7	8	8	8	8	8	7	8
Drosophila.melanogaster	8	8	7	8	8	8	8	8	7	8	8	8	8	8	8	7
Escherichia.coli	8	8	7	8	8	8	8	8	7	8	8	8	8	8	7	7
Helicobacter.pylori	8	8	7	8	8	8	8	8	8	7	8	8	8	8	7	7
Homo.Sapiens	8	8	8	8	8	8	8	8	8	8	8	8	8	8	8	8
Leishmania.major	8	8	7	8	8	8	8	8	7	8	8	8	8	8	8	7
M.Xanthus	8	8	6	8	8	8	8	8	7	8	8	8	8	8	6	8
Methanosarcina	8	8	8	8	8	8	8	8	8	8	8	8	8	8	8	8
MusMusculus	7	8	6	8	8	8	8	8	7	8	8	8	8	8	7	8
Myxococcus	8	8	6	8	8	8	8	8	7	8	8	8	8	8	6	8
OryzaSativa	8	8	8	8	8	8	8	8	8	8	8	8	8	8	8	8
P.Horikoshii	8	8	8	8	8	8	8	8	8	8	8	8	8	8	8	8
Plasmodiumfalciparum3D7	7	7	6	8	8	8	8	8	7	8	8	8	8	8	8	8
Pyrococcus	8	8	8	8	8	8	8	8	8	8	8	8	8	8	8	8
Schizosaccharomyces.Pombe	8	8	7	8	8	8	8	8	8	8	8	8	8	7	7	7
Staphylococcus.aureus	7	7	6	8	8	7	7	7	7	8	8	8	8	8	8	8
Streptomyces.coelicolorA3	8	8	8	8	8	8	8	8	8	8	8	8	8	8	7	8
Sulfolobus.solfataricus	8	8	7	8	8	8	8	8	8	8	8	8	8	8	8	8
Thermoplasma.acidophilum	8	8	7	8	8	8	8	8	8	8	8	8	8	8	8	7
ZeaMays	8	8	8	8	8	8	8	8	8	8	8	8	8	8	8	8

Table 7. FRAME +1: relative coverage rank over 25 genomes for the 16 codes identified as worst codes. No matter the organism, such codes are almost invariably ranked eighth within their equivalence class. They are obtained as the Keto-Amino transformation of the best codes.

$\alpha_2(\cdot)$	X_{173}	X_{23}	X_{98}	X_{166}	X_4	X_{172}	X_{21}	X_{24}	X_{97}	X_{171}	X_3	X_{165}	X_{115}	X_{161}	X_{41}	X_{107}
AeropyrumPernix	1	1	2	1	1	1	1	1	1	1	1	1	1	1	1	1
Arabidopsis.Thaliana	1	1	2	1	1	1	1	1	2	1	1	1	1	1	1	1
Archaeoglobus	1	1	2	1	1	1	1	1	2	1	1	1	1	1	1	1
Bacillus.subtilis	1	1	1	1	1	1	1	1	1	1	1	1	1	1	2	1
Caenorhabditis.elegans	1	1	2	1	1	1	1	1	2	1	1	1	1	1	1	1
DanioRerio	1	1	2	1	1	1	1	1	2	1	1	1	1	1	1	1
Drosophila.melanogaster	1	1	3	1	1	1	1	1	2	1	1	1	1	1	1	1
Escherichia.coli	1	1	1	1	1	1	1	1	1	1	1	1	1	1	1	1
Helicobacter.pylori	1	1	1	1	1	1	1	1	1	1	1	1	1	2	2	2
Homo.Sapiens	1	1	2	1	1	1	1	1	2	1	1	1	1	1	1	1
Leishmania.major	1	1	2	1	1	1	1	1	2	1	1	1	1	1	1	1
M.Xanthus	2	1	2	1	1	1	1	1	2	1	1	1	1	1	1	1
Methanosarcina	1	1	2	1	1	1	1	1	1	1	1	1	1	1	1	1
MusMusculus	1	1	2	1	1	1	1	1	2	1	1	1	1	1	1	1
Myxococcus	2	1	2	1	1	1	1	1	2	1	1	1	1	1	1	1
OryzaSativa	1	1	2	1	1	1	1	1	2	1	1	1	1	1	1	1
P.Horikoshii	1	1	1	1	1	1	1	1	1	1	1	1	1	1	1	1
Plasmodiumfalciparum3D7	1	1	1	1	1	1	1	1	1	1	2	2	1	1	2	1
Pyrococcus	1	1	1	1	1	1	1	1	1	1	1	1	1	1	1	1
Schizosaccharomyces.Pombe	1	1	1	1	1	1	2	1	1	1	2	1	1	1	2	1
Staphylococcus.aureus	1	1	1	1	1	1	1	1	1	1	2	2	2	2	2	2
Streptomyces.coelicolorA3	2	1	2	1	1	2	1	1	2	1	1	1	1	1	1	3
Sulfolobus.solfataricus	1	1	1	1	1	1	1	1	1	1	1	1	1	1	1	2
Thermoplasma.acidophilum	2	1	2	1	1	1	1	1	2	1	1	1	1	1	1	1
ZeaMays	1	1	2	1	1	1	1	1	2	1	1	1	1	1	1	1

Table 8. FRAME +2: relative coverage rank over 25 genomes for the 16 codes identified as best codes. No matter the organism, such codes are almost invariably ranked first within their equivalence class.

$\alpha_2(\cdot)$	X_{192}	X_{87}	X_{53}	X_{191}	X_{86}	X_{195}	X_{91}	X_{57}	X_{54}	X_{208}	X_{90}	X_{194}	X_{136}	X_{207}	X_{93}	X_{146}
AeropyrumPernix	8	8	8	8	8	8	8	8	8	8	8	8	8	8	8	8
Arabidopsis.Thaliana	8	8	7	8	8	8	8	8	7	8	8	8	8	8	7	7
Archaeoglobus	8	8	8	8	8	8	8	8	8	8	8	8	8	8	8	7
Bacillus.subtilis	8	8	8	8	8	8	8	8	8	8	8	8	8	8	8	7
Caenorhabditis.elegans	8	8	7	8	8	8	8	8	7	8	8	8	8	8	8	8
DanioRerio	8	8	7	8	8	8	8	8	7	8	8	8	8	8	8	8
Drosophila.melanogaster	7	8	7	8	8	8	8	8	7	8	8	8	8	8	8	7
Escherichia.coli	8	8	7	8	8	8	8	8	7	8	8	8	8	8	8	6
Helicobacter.pylori	8	8	7	8	8	8	8	8	7	7	8	8	8	8	8	8
Homo.Sapiens	8	8	7	8	8	8	8	8	8	8	8	8	8	8	8	8
Leishmania.major	8	8	7	8	8	8	8	8	7	8	8	8	8	8	8	6
M.Xanthus	8	8	6	8	8	8	8	8	7	8	8	8	8	8	8	8
Methanosarcina	8	8	7	8	8	8	8	8	7	8	8	8	8	8	8	8
MusMusculus	7	8	6	8	8	7	8	7	7	8	8	8	8	8	7	8
Myxococcus	8	8	6	8	8	8	8	8	7	8	8	8	8	8	8	8
OryzaSativa	8	8	7	8	8	8	8	8	8	8	8	8	8	8	8	8
P.Horikoshii	8	8	7	8	8	8	8	8	8	8	8	8	8	8	8	8
Plasmodiumfalciparum3D7	7	7	6	8	8	8	8	8	7	8	8	8	8	8	8	8
Pyrococcus	8	8	8	8	8	8	8	8	8	8	8	8	8	8	8	8
Schizosaccharomyces.Pombe	8	8	7	8	8	8	8	8	7	8	8	8	8	8	7	6
Staphylococcus.aureus	7	7	6	8	8	7	7	7	6	7	8	8	8	8	8	8
Streptomyces.coelicolorA3	8	8	8	8	8	8	8	8	8	8	8	8	8	8	8	8
Sulfolobus.solfataricus	8	8	7	8	8	8	8	8	8	8	8	8	8	8	8	8
Thermoplasma.acidophilum	8	8	8	8	8	8	8	8	8	8	8	8	8	8	8	7
ZeaMays	8	8	7	8	8	8	8	8	8	8	8	8	8	8	8	8

Table 9. FRAME +2: relative coverage rank over 25 genomes for the 16 codes identified as worst codes. No matter the organism, such codes are almost invariably ranked eighth within their equivalence class. They are obtained as the Keto-Amino transformation of the best codes.

Let Z_c and Z_r be the Fisher transformation of ρ_c and ρ_r, respectively, i.e.:

$$Z_c = \frac{1}{2}\ln\frac{1+\rho_c}{1-\rho_c}\text{, and } Z_r = \frac{1}{2}\ln\frac{1+\rho_r}{1-\rho_r}$$

and $\hat{Z}_c$, $\hat{Z}_r$ be the corresponding estimators when ρ_c and ρ_r are replaced by their sample analogue $\hat{\rho}_c$ and $\hat{\rho}_r$. The test statistic is

$$T = (n_1-3)(\hat{Z}_c-\bar{Z})^2 + (n_2-3)(\hat{Z}_r-\bar{Z})^2,$$

with $n_1 = 27$, $n_2 = 1000$ and

$$\bar{Z} = \frac{(n_1-3)\hat{Z}_c + (n_2-3)\hat{Z}_r}{n_1+n_2-6}.$$

Under H_0, T is distributed as a χ^2 random variable with 1 degree of freedom[3]. The test statistic results $T = 63.26$ and leads to rejecting the null hypothesis with a p-value $= 1.8e-15$.

2.5 Circular codes and codon influence on protein expression

We have tested the significance of the result by comparing the correlation coefficient obtained on the circular codes $\hat{\rho}_c = 0.847$ with those obtained on the random codes $\hat{\rho}_r = 0.439$. The test is the same as above and the test statistic results $T = 124.35$, with a p-value $= 7.1e-29$. Indeed, there is no strong correlation between single codon influence and single codon usage (Figure 2).

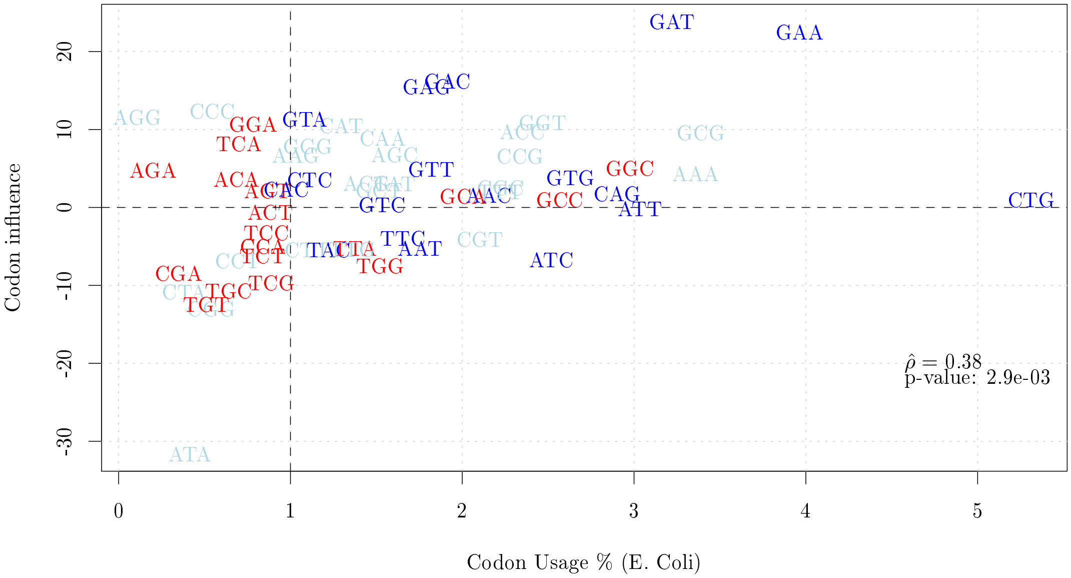

Figure 2. Codon usage in E. coli (percent) versus codon influence. The codons coloured in blue and in red belong to circular codes X_{173} and X_{192}, respectively. They are the best and worst codes within the set of 8 codes forming the equivalence class shown in Table 4 of the main article.

2.6 Circular codes and Ribosome Residence Time

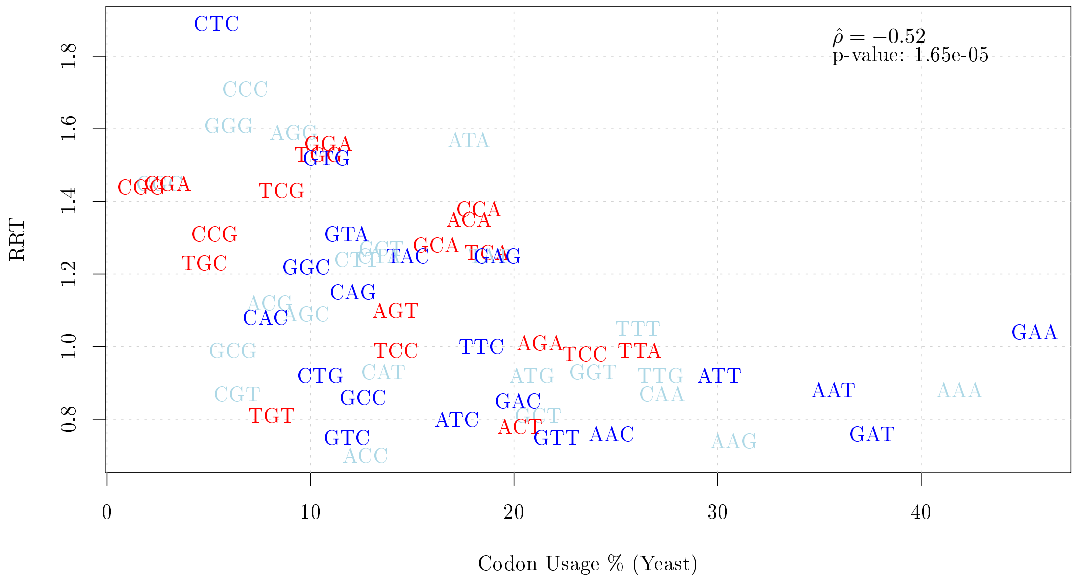

Figure 3. Codon usage in Yeast (percent) versus codon Ribosome Residence Time. The codons coloured in blue and in red belong to circular codes X_{173} and X_{192}, respectively. They are the best and worst codes within the set of 8 codes forming the equivalence class shown in Table 4 of the main article.

2.7 Circular code motifs are absent in the mRNA 5'-head and 3'-tail sequences

If circular code properties have a role in translation, then a differential coverage of the codons belonging to circular codes could apply as a function of position in the coding sequence. In Figure 4 we plotted the coverage of codes X_{173} (blue solid line) and X_{192} (red solid line) over rolling windows of 5 codons, computed over the first 100 codons of each complete coding sequence of the 25 organisms described in Table 3. Remarkably, both for code X_{173} and X_{192} there is a transient initial span of around 40 codon positions after which the rolling coverage over 5 codons reaches the value of the global coverage over the entire genome and fluctuates around it. While for code X_{173} the rolling coverage for the first positions is always lower than the global coverage, the rolling coverage for code X_{192} starts at a higher level with respect to the global coverage and decreases towards it. This appears to be a universal feature shared by all the organisms. The same is true for rolling windows up to 30 codons with no significant differences. The effect of the total codon content in the tail of the sequence was also reported to be influential[4] on expression. Accordingly, we also observed a universal tail effect in the coverage of coding sequences (Figure 5).

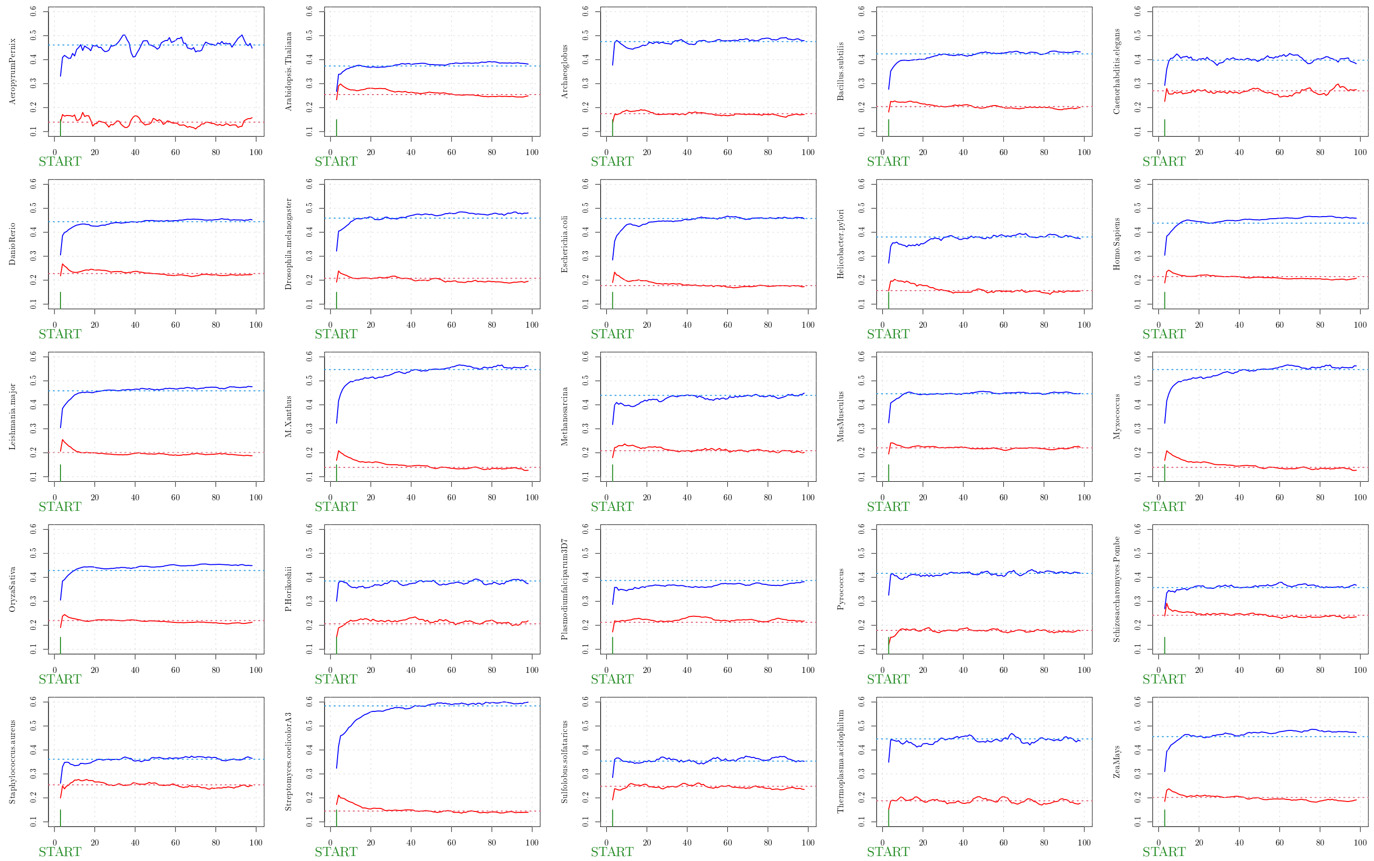

Figure 4. Rolling coverage computed on the first 100 codon positions, averaged over the whole set of complete coding sequences of 25 genomes. The blue and red solid lines correspond to code X_{173} and X_{192}, respectively. The dotted lines correspond to the global coverage of the codes over the whole genome.

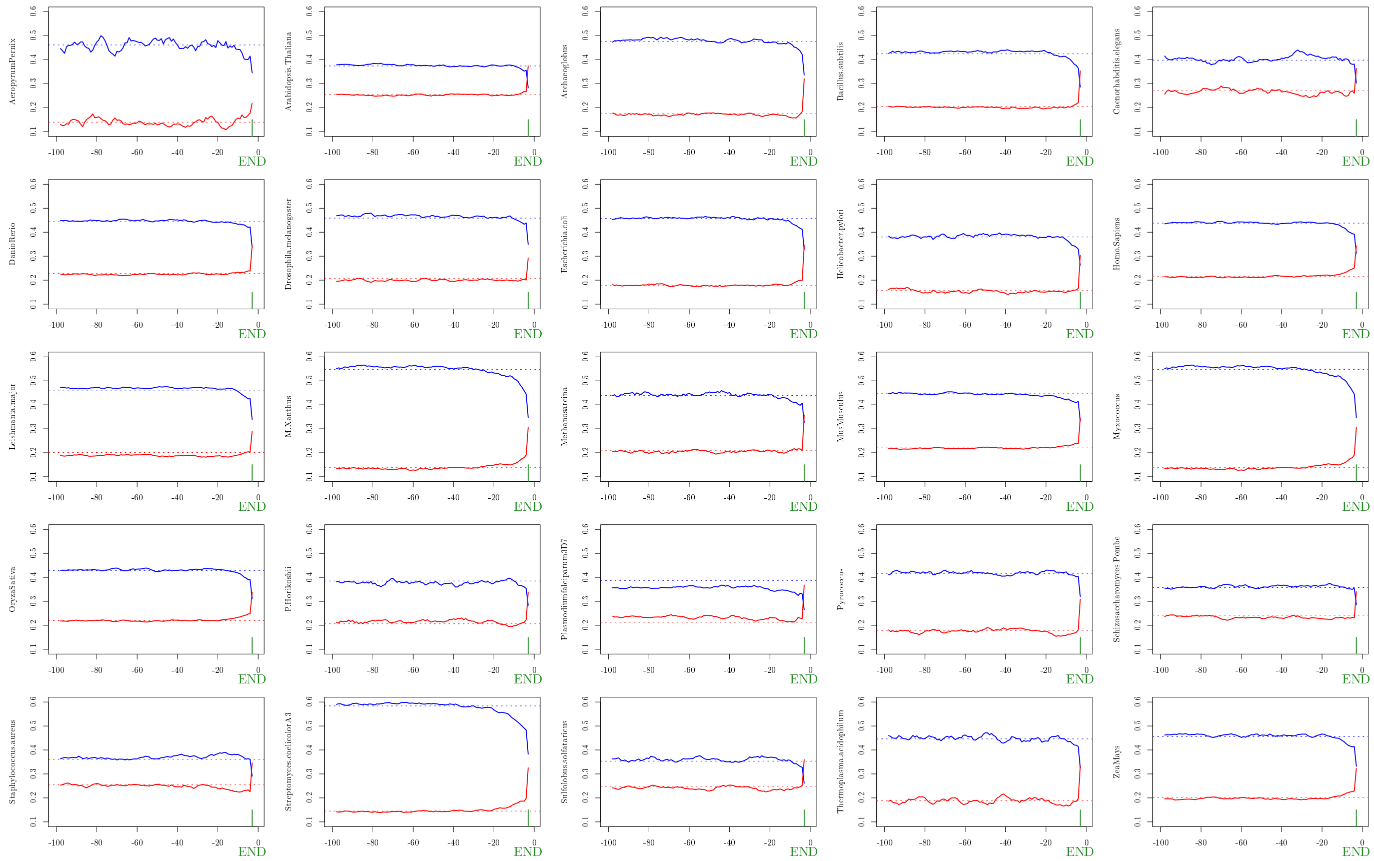

Figure 5. Rolling coverage computed on the last 100 codon positions, averaged over the whole set of complete coding sequences of 25 genomes. The blue and red solid lines correspond to code X_{173} and X_{192}, respectively. The dotted lines correspond to the global coverage of the codes over the whole genome.

2.8 Circular code properties correlate with the S/W character of the first two nucleotides of the codon

In Figure 6 we show the average frequencies of SWN, WWN, SSN and WSN codons for the group of codes with best coverage (blue) and worst coverage (red). The area of the bubbles is proportional to the average influence of each group of codons. Clearly, codons of the kind SWN and WWN identify the best codes, i.e. those associated to a higher expression level and coverage. Conversely, codons of the kind SSN and WSN characterize the codes having lower expression level and coverage.

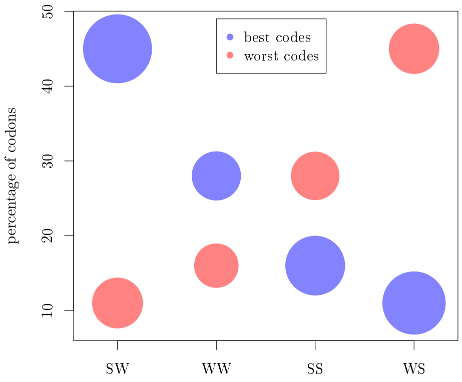

Figure 6. Comparison of codon composition of best codes (blue) and worst codes (red) according to the S/W chemical dichotomy of the first two nucleotides of the codon. The area of the bubbles is proportional to the average codon influence.

3 Discussion: circular code coverage correlates with mRNA stability

The codon stabilization coefficient (CSC) is a codon metric computed as the correlation between codon frequency in transcripts and the stability of the mRNA, experimentally measured through its half-life. In[5] the CSC is used to correlate mRNA half-life to the protein translation efficiency. In the following we correlate the circular code coverage with the CSC (the data are taken from Figure 4A of[5]). The results are presented in Figure 7, and can be interpreted similarly to Figures 3 and 4 of the main text.

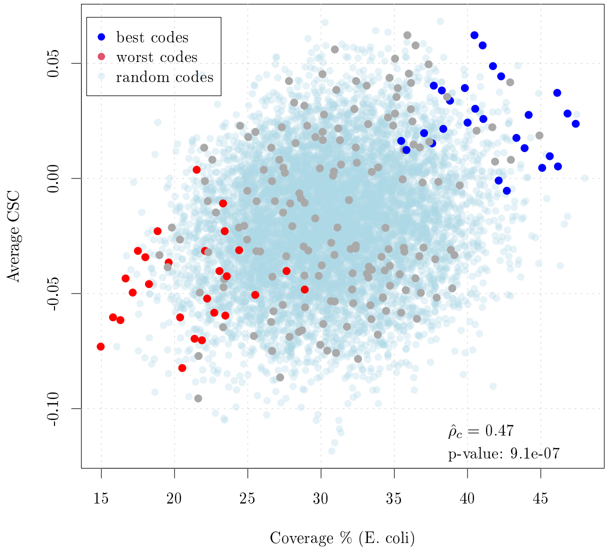

Figure 7. Average CSC (codon stabilization coefficient) versus Coverage (percent) computed on the 216 circular codes partitioned in 27 equivalence classes of 8 codes each. The points in blue and red correspond to the 27 best and 27 worst codes within their associated equivalence class, respectively. As for the speed of translation (Figure 3), the coverage is a predictor of codon influence and the best and worst codes within their equivalence class clusterize. The results for 8000 random codes are also shown in light blue and the p-value for the test that the observed correlation is equal to that of random codes is reported.

Consistently with the previous findings, circular codes properties positively correlate with the average codon stabilization coefficient within codes. The p-value = 9.1e-7 leads to rejecting the null hypothesis that the observed correlation (0.47) can be produced by random codes. The best and worst covering circular codes (blu and red, resp.) present a distinctive CSC that separates them from random codes. Note that such correlation is not present at the level of single codon usage, see Figure 8.

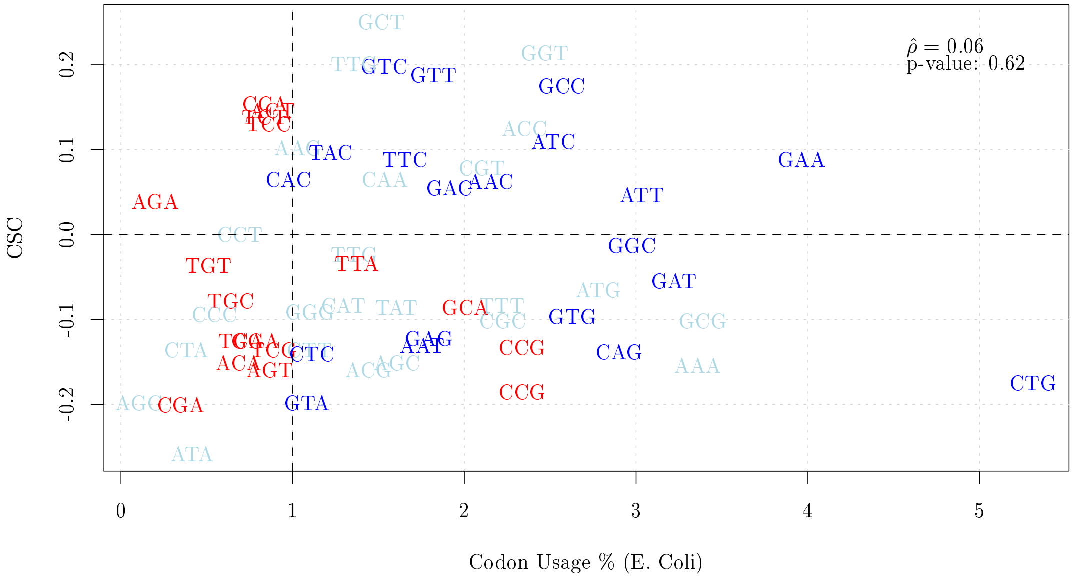

Figure 8. Codon usage in E. coli (percent) versus CSC (codon stabilization coefficient). The codons coloured in blue and in red belong to circular codes X_{173} and X_{192}, respectively. They are the best and worst codes within the set of 8 codes forming the equivalence class shown in Table 4 of the main article.

4 Circular codes and comma free codes

As detailed in Section 4 of the main article, the 216 self-complementary, C^3 circular codes can be partitioned in 27 classes according to the 8 symmetry transformations of the dihedral group (see Table 3 of the main article). In Table 11 we show such partition where the row numbers in bold indicate a class for which the codes corresponding to the identity (I, first column) and to the Keto-Amino transformation (KM, last column) have no common codons (they are disjoint)

	I	(AT)	(CG)	SW	YR	(ACTG)	(AGTC)	KM
**1**	173	176	203	206	183	182	193	192
**2**	23	33	77	81	13	37	65	87
**3**	98	10	96	8	52	45	55	53
4	25	35	76	85	50	47	59	56
5	20	34	75	80	17	40	69	89
**6**	166	216	164	213	186	187	189	191
**7**	4	104	6	102	16	42	61	86
8	27	30	72	84	38	12	88	64
9	117	160	118	157	130	131	133	135
10	111	159	116	151	119	126	138	145
11	22	29	71	79	2	1	100	99
**12**	172	175	202	205	181	184	196	195
**13**	21	31	74	78	11	39	68	91
**14**	24	32	73	83	49	48	60	57
**15**	97	9	95	7	51	46	58	54
**16**	171	174	201	204	167	178	200	208
**17**	3	103	5	101	15	43	62	90
**18**	165	215	163	212	185	188	190	194
19	26	28	70	82	36	14	92	66
20	123	124	141	143	105	106	150	147
**21**	115	158	113	155	129	132	134	136
**22**	161	214	162	211	168	179	197	207
23	122	125	140	142	110	108	152	149
**24**	41	94	18	67	19	44	63	93
**25**	107	156	112	148	120	127	139	146
26	177	210	169	199	170	180	198	209
27	137	121	144	128	114	109	153	154

Table 11. List of the 216 maximal, self-complementary, C^3 circular codes partitioned into 27 equivalence classes. Each class contains 8 codes linked through the transformations of the dihedral group D_8. We highlighted in bold the 16 classes for which the codes corresponding to the identity (I, first column) and to the Keto-Amino transformation (KM, last column) have no common codons (they are disjoint).

References

1. Nakamura, Y., Gojobori, T. & Ikemura, T. Codon usage tabulated from the international DNA sequence databases. *Nucleic Acids Res.* **25**, 244, DOI: 10.1093/nar/25.1.244 (1997).
2. El Soufi, K. & C.J., M. Circular code motifs in genomes of eukaryotes. *J. Theor. Biol.* **408**, 198 – 212, DOI: https://doi.org/10.1016/j.jtbi.2016.07.022 (2016).
3. Paul, S. Test for the equality of several correlation coefficients. *The Can. J. Stat. / La Revue Can. de Stat.* **17**, 217–227 (1989).
4. Boël, G. *et al.* Codon influence on protein expression in E. coli correlates with mRNA levels. *Nature* **529**, 358 – 376, DOI: https://doi.org/10.1038/nature16509 (2016).
5. Carneiro, R., Requião, R., Rossetto, S., Domitrovic, T. & Palhano, F. Codon stabilization coefficient as a metric to gain insights into mRNA stability and codon bias and their relationships with translation. *Nucleic Acids Res.* **47**, 2216–2228, DOI: 10.1093/nar/gkz033 (2019). https://academic.oup.com/nar/article-pdf/47/5/2216/28041785/gkz033.pdf.
